# Leveraging stacked classifiers for exploring the role of hedonic processing between major depressive disorder and schizophrenia

**DOI:** 10.1017/S0033291725101207

**Published:** 2025-07-23

**Authors:** Yating Huang, Jiayu He, Xinyue Zhang, Ji Chen, Zhenghui Yi, Qinyu Lv, Chao Yan

**Affiliations:** 1 School of Psychology and Cognitive Science, East China Normal University, Shanghai, China; 2 Center for Brain Health and Brain Technology, Global Institute of Future Technology, Shanghai Jiao Tong University, Shanghai, China; 3 Shanghai Mental Health Center, Shanghai Jiao Tong University School of Medicine, Shanghai, China; 4 Key Laboratory of Philosophy and Social Science of Anhui Province on Adolescent Mental Health and Crisis Intelligence Intervention, Hefei Normal University, Hefei, China

**Keywords:** anhedonia, machine learning, major depressive disorder, reward processing, schizophrenia, stacking model

## Abstract

**Background:**

Anhedonia, a transdiagnostic feature common to both Major Depressive Disorder (MDD) and Schizophrenia (SCZ), is characterized by abnormalities in hedonic experience. Previous studies have used machine learning (ML) algorithms without focusing on disorder-specific characteristics to independently classify SCZ and MDD. This study aimed to classify MDD and SCZ using ML models that integrate components of hedonic processing.

**Methods:**

We recruited 99 patients with MDD, 100 patients with SCZ, and 113 healthy controls (HC) from four sites. The patient groups were allocated to distinct training and testing datasets. All participants completed a modified Monetary Incentive Delay (MID) task, which yielded features categorized into five hedonic components, two reward consequences, and three reward magnitudes. We employed a stacking ensemble model with SHapley Additive exPlanations (SHAP) values to identify key features distinguishing MDD, SCZ, and HC across binary and multi-class classifications.

**Results:**

The stacking model demonstrated high classification accuracy, with Area Under the Curve (AUC) values of 96.08% (MDD versus HC) and 91.77% (SCZ versus HC) in the main dataset. However, the MDD versus SCZ classification had an AUC of 57.75%. The motivation reward component, loss reward consequence, and high reward magnitude were the most influential features within respective categories for distinguishing both MDD and SCZ from HC (*p* < 0.001). A refined model using only the top eight features maintained robust performance, achieving AUCs of 96.06% (MDD versus HC) and 95.18% (SCZ versus HC).

**Conclusion:**

The stacking model effectively classified SCZ and MDD from HC, contributing to understanding transdiagnostic mechanisms of anhedonia.

## Introduction

Anhedonia, a common symptom across various mental disorders, including Major Depressive Disorder (MDD) and Schizophrenia (SCZ) (Pelizza & Ferrari, 2009), is associated with poorer premorbid functioning, diminished social competence, and adverse long-term outcomes. This symptom encompasses a range of abnormalities in hedonic processing (Borsini et al., 2020). However, previous research has often overlooked the multifaceted nature of anhedonia, limiting its potential as a transdiagnostic marker for understanding and differentiating between MDD and SCZ (Rizvi, Pizzagalli, Sproule, & Kennedy, 2016). Since hedonic processing is a key psychopathological feature of anhedonia, emphasizing its multidimensional aspects could offer deeper insights into anhedonia as a transdiagnostic construct (Kring & Barch, 2014; Su & Si, 2022).

A growing body of research has employed machine learning (ML) to identify complex patterns, offering new opportunities for classifying SCZ and MDD (Yang, Xing, Yang, & Gong, 2022). However, reported classification performances vary substantially, with AUC values ranging from 74% to 94.73% for SCZ and from 54% to 94.6% for MDD (Meinke, Lueken, Walter, & Hilbert, 2024; Quaak, Van De Mortel, Thomas, & Van Wingen, 2021). Such variability may be due to the reliance on a heterogeneous set of features–spanning clinical, demographic, and biological domains–that are not specifically grounded in symptom constructs. However, few studies have specifically focused on anhedonia-related features for classification. To date, only one ML study utilized imaging data to isolate hedonic-processing features, achieving high classification accuracy for SCZ (Koch et al., 2015). Notably, neglecting the multidimensional nature of hedonic processing may undermine the robustness and reliability of classification performance (Treadway & Zald, 2013). Since hedonic processing encompasses distinct components, consequences, and magnitudes (Meadows, Gable, Lohse, & Miller, 2016), integrating these nuanced aspects could provide a more comprehensive insight into the complex hedonic processing mechanisms.

Kring and Barch (2014) introduced the Temporal Experience of Pleasure (TEP) framework, which categorizes the hedonic processing into: anticipatory pleasure (incorporating both ‘prediction’ and ‘feeling’ components), consummatory pleasure, remembered pleasure, and motivation. Evidence suggests that MDD patients experience deficits in both anticipatory and consummatory pleasures (Geugies et al., 2022), whereas individuals with SCZ show comparable levels of consummatory pleasure but reduced motivation and anticipatory pleasure (Wang et al., 2023). Studies on MDD indicate significant impairments in the ‘feeling’ component, where individuals experience blunted emotional responses to anticipated rewards, diminishing their engagement with potentially rewarding stimuli (Treadway & Zald, 2011; Whitton et al., 2016). In contrast, research on SCZ highlights a cognitive basis for anhedonia, revealing that SCZ patients experience impairments in the predictive dimension of anticipatory pleasure, as well as a dissociation between anticipatory pleasure and motivation (Boka et al., 2020). The distinction between MDD and SCZ emphasizes differing impairments in anticipatory pleasure, underscoring the need to examine these components to better understand the heterogeneity of anhedonia.

Research consistently demonstrates that reward consequences and magnitudes play a critical role in shaping findings on hedonic impairments in both MDD and SCZ. For instance, patients with SCZ often show asymmetrical processing of reward consequences, exhibiting impaired learning from rewards and relatively intact learning from punishments (Boka et al., 2020). The Emotion Context Insensitivity (ECI) theory posits that MDD patients have blunted emotional responses to both pleasurable and aversive stimuli, limiting their ability to experience a full range of emotions. Additionally, studies indicate that MDD and SCZ exhibit distinct responses to high-magnitude rewards (Wang et al., 2015). Patients with MDD struggle to distinguish between gain and non-gain consequences during reward anticipation (Knutson et al., 2008; Smoski, Rittenberg, & Dichter, 2011), whereas individuals with SCZ face difficulties adapting their behavior based on reward feedback, particularly in high-reward contexts (Wang et al., 2023). These findings highlight the need for further investigation into the distinct manifestations of complex reward components in MDD and SCZ and how these differences could improve diagnostic accuracy.

Previous studies employing traditional ML algorithms often relied on multiple modalities of features for generalized classification. To improve predictive modeling, the stacking ensemble method (stacking) leverages the strengths of multiple models, enhancing performance and capturing complex data patterns (Vergaray, 2023). This study aimed to determine the optimal ML model for classifying MDD and SCZ from healthy controls (HC) within the TEP framework, exploring the contributions of hedonic processing features, including components, consequences, and magnitudes. We also assessed model performance across various feature sets to evaluate the impact of feature simplicity. We hypothesized that: (1) the stacking model would achieve high accuracy (ACC) and AUC, demonstrating the utility of reward processing dysfunction in distinguishing MDD and SCZ from HC, as well as revealing disease-specific patterns between MDD and SCZ; (2) within the ‘Anticipatory Pleasure’ dimension, the ‘Feeling’ deficit would be key for MDD and “Prediction” for SCZ classification, and high-magnitude monetary rewards in positive outcome contexts (i.e. monetary gains) would be the most influential feature sets; (3) models with simplified feature would maintain acceptable classification performance.

## Methods

### Participants

A total of 99 participants with MDD, 100 with SCZ were recruited from Shanghai Mental Health Center (SMHC), Beijing Anding Hospital, Capital Peak Hospital, and Fuzhou Mental Health Center. Additionally, 113 HCs were enrolled from East China Normal University and local communities in Shanghai, China. For dataset allocation, 56 MDD participants and 64 HCs from SMHC were used to form independent training datasets, while the remaining MDD patients and HCs were assigned to testing datasets. Similarly, 54 SCZ participants from SMHC and 61 HCs were assigned to the training datasets, while the remaining SCZ patients and HCs were allocated to the testing datasets (See Table 1). To address site-specific bias and evaluate generalizability, we employed fixed-site training (FST) and random-site training (RST). FST used data from SMHC for training and other sites for testing, while RST pooled data from all sites and applied random splits.

**Table 1.** Demographic of participants’ socio-demographic information

	MDD(N = 99) M ± SD	SCZ(N = 100) M ± SD	HC(N = 113) M ± SD	*F*	*p*
Gender (Female%)	79.80%	57.00%	61.95%	6.632	0.002
Age (Years)	25.25 ± 7.19	27.58 ± 8.45	22.17 ± 5.22	15.916	<0.001
Education (Years)	14.85 ± 3.41	13.19 ± 2.67	14.39 ± 2.18	8.439	<0.001
BDI score (M ± SD)	18.27 ± 13.90	–	–		
PANSS_pos (M ± SD)	–	11.72 ± 4.88	–		
PANSS_neg (M ± SD)	–	14.79 ± 7.22	–		
PANSS_gen (M ± SD)	–	27.83 ± 9.27	–		
PANSS_Total (M ± SD)	–	54.34 ± 19.12	–		
Medication (FLX/CPZ mg/d)	35.20 ± 20.44	321.97 ± 255.25	–		

*Note:* BDI, ‘the beck depression inventory’; PANSS, ‘the positive and negative syndrome scale’; FLX, ‘fluoxetine’; CPZ, ‘chlorpromazine’.

In this study, 62 MDD and 92 SCZ participants provided medication data, and 76 SCZ participants provided PANSS data (Post-hoc ANOVA of socio-demographic info: Supplementary Table S1).

Data from 60 MDD patients were previously published, which explored emotion context insensitivity within the depression spectrum. Additionally, data from 44 SCZ patients were used in another published paper (Yan et al., 2019). All other data are original.

The inclusion criteria for this study were: (1) a diagnosis of MDD or SCZ based on DSM-IV criteria; (2) an age range of 12–55 years; and (3) the ability to understand and comply with study procedures. The exclusion criteria included: (1) a history of brain injury or neurological disorders; (2) the presence of other psychiatric disorders (including but not limited to anxiety disorders, bipolar disorder, post-traumatic stress disorder, obsessive-compulsive disorder, personality disorders, or any other disorder as per DSM-5 criteria); (3) a history of substance or alcohol abuse; and (4) having received electroconvulsive therapy within the past 3 months. All participants provided written informed consent and voluntarily agreed to participate. The study was approved by the University Committee on Human Research Protection at East China Normal University (HR 035-2018).

### Measures and feature extraction

#### Money incentive delay task

**Figure 1.**
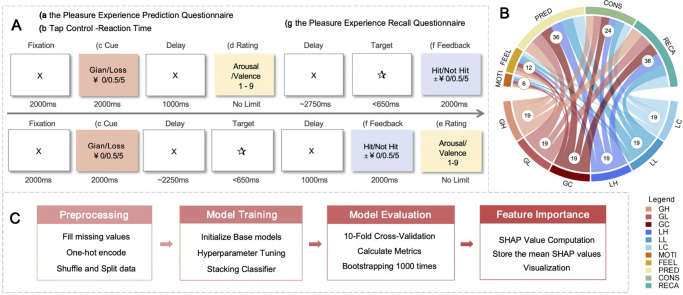
The flow of the feature extraction. *Note:* (A) MID Paradigm Scheme. (a) Reward component: Anticipatory pleasure – Prediction (PRED); (c) Reward consequences: Gain/Loss (G/L); Reward magnitudes: High/Low/Control (H/L/C); (d) Reward component: Anticipatory pleasure- Feeling (FEEL); (e) Reward component: Consummatory pleasure (CONS); (f-b) Reward component: Motivation (MOTI); (g) Reward component: Remembered pleasure (Recall-RECA). (B)Combination of Feature Sets: The number within each circle represents the count of features included in that feature set. Detailed descriptions of the features are provided in Supplementary Table S3. GH, ‘gain high’; GL, ‘gain low’; GC, ‘gain control’; LH, ‘loss high’; LL, ‘loss low’; LC, ‘loss control’. (C)The workflow of Machine Learning.

The Monetary Incentive Delay (MID) task assesses multiple aspects of hedonic processing. Participants’ baseline reaction times were first recorded during a simple task, where they pressed the space bar in response to a target (i.e. a star). The task consists of 72 trials, divided into four blocks: two anticipatory pleasure blocks and two consummatory pleasure blocks. In each trial, participants saw a cue indicating the type of trial (e.g. potential gain or loss) with reward magnitudes set at ¥0, ¥0.5, or ¥5. After the cue, a target appeared, and participants responded quickly to earn a reward or avoid a loss. Each trial ends with feedback on whether the target was hit and the monetary outcome. Throughout the blocks, participants rated their arousal and valence on a 9-point Likert scale during both the anticipation and feedback phases, reflecting emotional responses to reward. The task also tracks reaction times to targets as an indicator of motivation. Baseline reaction times (RTs) were subtracted from reward-condition RTs to minimize attentional and motor confounds, thereby isolating motivational processes (Winstanley et al., 2010). Additionally, participants completed the Pleasure Experience Prediction Questionnaire before the task and the Pleasure Experience Recall Questionnaire 30 minutes afterward, both using similar rating scales to assess the ‘prediction’ and ‘remembered pleasure’ (See Figure 1a for an overview of the MID task scheme).

#### Feature extraction

We extracted a total of 114 features from the MID task, organized into five key components: ‘prediction’ (PRED) and ‘feeling’ (FEEL) components of anticipatory pleasure (ANTI), consummatory pleasure (CONS), remembered pleasure (RECA), and motivation (MOTI). Reward consequences were categorized as gain (GAIN) or loss avoidance (LOSS) with three monetary magnitude conditions: HIGH, LOW, and CONTROL (no reward). These were combined to create six conditions: Gain/Loss High (GH, LH), Gain/Loss Low (GL, LL), and Gain/Loss Control (GC, LC) (See Figure 1b and Supplementary Table S2).

### Machine learning analyses

#### Data preprocessing

We began our analysis by excluding two MDD participants with feature values containing more than 10% outliers, defined as values exceeding three standard deviations from the mean, and any subjects with missing data exceeding 20% of the features. All remaining subjects met the inclusion criteria. Missing values were imputed with the mean of each feature’s column. Categorical variables were transformed using *one-hot encoding*, creating binary indicator columns for each category. The *train_test_split* function from *scikit-learn* was used to create an 80–20 training and testing in the dataset that did not have independent splits. We also performed *Standardization* and *Z-score normalization* on the models that are sensitive to these processes.

#### Machine learning models

In this study, we evaluated various ML models to classify participants based on behavioral responses within multidimensional reward processing. We selected ML models representing different classification principles, including Random Forest (RF), Decision Tree (DT), Logistic Regression (LR), K-Nearest Neighbors (KNN), Support Vector Machine (SVM), Gradient Boosting Trees (GBT), Naive Bayes (NB), and Extreme Gradient Boosting (XGB). Additionally, we developed a stacking that combined the best-performing models at the base level (level 0), with Logistic Regression (LR) serving as the meta-classifier at level 1 (details provided in Supplementary Table S3). To further validate model selection, we conducted a performance comparison between the stacking model and two deep learning models: MLP and TabNet (see Supplementary Note for implementation details and hyperparameter configurations).

We conducted hyperparameter tuning for models by using *Grid Search* with cross-validation. Each model was initially configured with default settings, and specific ranges of hyperparameters were defined. The *GridSearchCV* function was utilized to evaluate every possible combination of these hyperparameters through 10-fold cross-validation. This process involved training the model on various subsets of the data and assessing performance based on accuracy. The fit method was employed to systematically search for the optimal hyperparameters, testing all combinations within the specified ranges to select the best-performing configuration.

#### Feature importance analysis

We assessed feature importance to determine each feature’s contribution to classification accuracy. Multiple methods were employed across models (see Supplementary Table S3). Given the potential for multicollinearity and interactions between features, we opted not to rely on the absolute values of coefficients to assess feature importance in LR. Instead, we used the Variance Inflation Factor (VIF) to evaluate multicollinearity among features to investigate whether collinearity affected the classification performance. VIF quantifies the extent to which the variance of a predictor is inflated due to its correlation with other predictors. It was calculated as the R^2^ value from regressing the predictor on all other predictors. A VIF value exceeding 10 generally indicates significant multicollinearity, suggesting that the predictor may be redundant or highly collinear with other predictors. We evaluated eight ML models for MDD and SCZ classification using all 114 features. To assess multicollinearity effects, we compared performance before and after removing features with VIF > 10. No significant differences in accuracy or AUC were found for either classification (see Supplementary Tables S4–S6).

For the stacking model, SHapley Additive exPlanations (SHAP) values were calculated for each base model in level 0 using *‘shap.KernelExplainer’* for non-tree-based models and ‘shap.TreeExplainer’ for tree-based models. After computing SHAP values for each individual model, we aggregated them by calculating their arithmetic mean to obtain a comprehensive measure of feature importance across all models in the ensemble. This combined SHAP value offers a more robust and holistic assessment, capturing the contributions of each feature across the diverse modeling approaches used in the ensemble.

We performed 100 iterations of *ShuffleSplit* cross-validation, calculating SHAP values for each base model within the stacking framework during each iteration. To assess feature importance, we averaged the absolute SHAP values across all iterations, recording the mean importance scores for each feature. Features were then categorized into four dimensions and five components based on the TEP framework, as well as two reward consequences and three reward magnitudes. Given the varying number of features within each category (See Figure 1b), we ultimately compared the average importance scores across these feature sets to evaluate their relative contributions and ranked the features accordingly.

#### Model evaluation

We implemented a 10-fold cross-validation strategy using the *KFold* method to evaluate classifier performance on independent training and testing datasets. The model’s performance was assessed using confusion matrices, which allowed for the calculation of sensitivity and specificity for both the training and test sets. These metrics provide overall accuracy while assessing the precision-recall balance, critical for imbalanced datasets. Additionally, the AUC was employed to assess the model’s discriminative ability, providing a single scalar value summarizing the model’s performance. To ensure robustness, we bootstrapped the test set 1,000 times, calculating the AUC for each resample. The 95% confidence interval, determined by the 2.5th and 97.5th percentiles of the bootstrapped AUCs, offers insight into the range within which the true AUC is likely to fall, thereby providing a robust evaluation of the model’s generalization ability (see workflow in Figure 1c).

## Results

### Selection of optimal model

We evaluated the performance of eight ML models for separately classifying MDD and SCZ using all 114 features. The top-performing models included XGB, RF, LR, SVM, and GBT (see Supplementary Tables S7–S8; The binary classification performance of the RF and XGB models before and after regularization is presented in Supplementary Table S9 and Supplementary Figures S1–S2). Based on these results, we developed a stacking model with RF, GBT, SVM, and XGB as base learners at level 0 and LR as the meta-learner at level 1. This stacking model outperformed the individual models, achieving optimal performance with AUC_MDD versus HC_ = 97.04% and AUC_SCZ versus HC_ = 96.05%. Its performance was also benchmarked against the MLP and TabNet deep learning models, with the stacking model achieving higher AUCs in both classification tasks (see Supplementary Table S10 for full results).

To evaluate the model’s generalizability, we trained it on data from an independent training set and validated it on a multicenter testing set from other sites. The model demonstrated consistent and robust performance across these diverse datasets, with AUC_MDD versus HC_ = 96.08% and AUC_SCZ versus HC_ = 91.77%. Additionally, we observed a decline in model performance when classifying between MDD and SCZ, with an AUC_MDD versus SCZ_ = 57.75% (p = 0.775) (See confusion matrix of MDD versus SCZ in Supplementary Figure S3). The performance in three-group classification (MDD, SCZ, and HC) was similarly limited, with an AUC_MDD versus SCZ versus HC_ = 66.75% (Please see Table 2). We also evaluated the stacking model using data pooled across all fixed sites (FST) and with random sites classification (RST), where training and test sets were split by site (see Table 2). While site-specific splitting led to a slight performance decline, the model maintained high generalizability, with AUCs above 90% for distinguishing patient groups from HC.

**Table 2.** Dichotomous and trichotomous performance of stacking model

	ACC	SENS	SPEC	AUC
				(2.5–97.5%percentiles)
MDD versus HC (FST)	88.40%	100.00%	82.80%	97.04% (91.48%–100.00%)
MDD versus HC (RST)	83.82%	83.87%	83.87%	96.08% (88.89%–100.00%)
SCZ versus HC (FST)	88.40%	94.70%	79.20%	96.05% (89.29%–100.00%)
SCZ versus HC (RST)	82.47%	71.74%	92.16%	91.77% (82.81%–98.51%)
MDD versus SCZ (FST)	47.50%	45.00%	50.00%	57.75% (39.14%–75.19%)
MDD versus SCZ (RST)	28.57%	32.61%	22.58%	24.40% (10.43%–41.85%)
MDD versus SCZ versus HC (FST)	63.49%	45.00%	75.00%	66.75% (50.79%–74.60%)
MDD versus SCZ versus HC (RST)	65.62%	68.18%	96.00%	86.25% (79.45%–91.90%)

*Note:* FST, ‘fixed site training’; RST, ‘random site training’; ACC, ‘accuracy’; SENS, ‘sensitivity’; SPEC, ‘specificity’; AUC, ‘area under the curve’.

To evaluate demographic confounds, we ran 1,000 iterations of stacking models using reward features alone versus models additionally including age, gender, or education. Bonferroni-corrected t-tests on AUCs showed no effect for SCZ versus HC. For MDD versus HC, age had no impact, but education and gender significantly reduced AUC (*p <* 0.001), indicating potential confounding (see Supplementary Table S11). Our model also exhibited strong and consistent performance in distinguishing patients from healthy controls across varying levels of symptom severity and medication status (see Supplementary Tables S12–S13 for details).

### Feature importance

We extracted SHAP values for MDD and SCZ from the RF, XGB, and GBT models within the two binary stacking models (MDD versus HC and SCZ versus HC). Based on different components of hedonic processing, we visualized the impairments in MDD and SCZ across distinct features using mirrored beeswarm plots (see Supplementary Figures S4–S8).

The feature of MOTI consistently held a significantly higher average importance weight than other components in both MDD versus HC (*F*(4, 490) = 17954.088, *p* < .001, 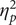 =.993) and SCZ versus HC classifications (*F*(4,495) = 49776.762, *p* < .001, 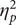 =.999). Within the anticipatory pleasure dimension, an independent samples t-test revealed significant differences in feature importance between the FEEL and PRED components for MDD versus HC and SCZ versus HC classifications. Specifically, the FEEL component exhibited a higher importance weight in classifying MDD from HC (*t*(200) = 130.269, *p* < .001, *Cohen’s d* = 18.331), whereas the PRED component showed greater importance weight in classifying SCZ versus HC (*t*(200) = −79.672, *p* < .001, Cohen’s *d* = −11.211).

**Figure 2.**
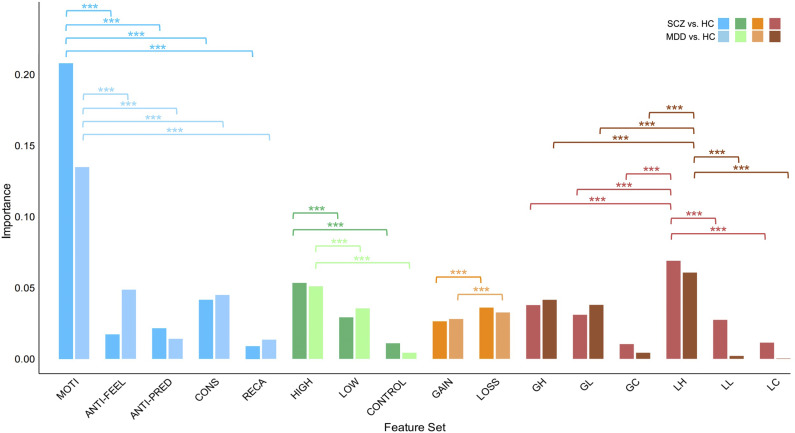
Importance of each feature set by stacking. Note: MDD, ‘major depressive disorder’; SCZ, ‘schizophrenia’; HC, ‘healthy controls’; MOTI, ‘motivation’; ANTI-FEEL, ‘feeling’; ANTI-PRED, ‘prediction’; CONS, ‘consummatory pleasure’; RECA, ‘remembered pleasure’; GAIN, ‘gain reward’; LOSS, ‘avoid loss reward’; GH, ‘gain high’; GL, ‘gain low’; GC, ‘gain control (control, no rewards)’; LH, ‘loss high’; LL, ‘loss low’; LC, ‘loss control’.

In terms of reward consequences, the LOSS feature exhibited significantly higher importance weight than the GAIN feature in both MDD versus HC (*t*(196) = −39.664, *p* < .001, Cohen’s *d =* −5.582) and SCZ versus HC classification (*t*(198) = −52.586, *p* < .001, Cohen′s *d* = −7.400). Regarding reward magnitudes, the HIGH magnitude feature was more influential in both classifications (MDD versus HC: *F*(2,294) = 128615.675, *p* < .001, 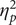 *=* 0.998; SCZ versus HC: *F*(2, 297) = 80048.145, *p* < .001, 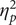 *=*1.000). Notably, when feature sets were categorized into GH, GL, GC, LH, LL, and LC by combining reward consequences and magnitudes, the LH feature set emerged as the most influential in both MDD versus HC (*F*(5,588) = 28361.939, *p* < .001, 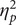 = .993) and SCZ versus HC (*F*(5,594) = 49776.762, *p* < .001, 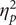 = .998). (See Figure 2 for the importance of each feature set; Detailed statistics are provided in Supplementary Tables S14–S22; Feature set importance for other algorithms can be found in Supplementary Tables S23–S24).

### Evaluating classification performance with simplified features

We selected the following feature sets for further analysis: (1) the MOTI Set (k = 6 features) and ANTI Set (*k* = 12 features); (2) the LOSS Set (*k* = 57 features); (3) the HIGH Set (*k* = 38 features); (4) the TOP 8 Co-emerging Factors (*k* = 8 features), representing the eight factors that ranked among the top 20 in importance for classifying MDD versus HC and SCZ versus HC. Feature reduction was applied based on these sets, and the stacking model was subsequently re-evaluated for MDD versus HC and SCZ versus HC classification. Notably, the results revealed that using a total of eight co-occurring factors, which ranked in the top 20 for both MDD versus HC (AUC = 95.29%) and SCZ versus HC (AUC = 91.73%), yielded the best performance for feature reduction (see Supplementary Table S25 for feature importance of the Top 20 features in MDD versus HC and SCZ versus HC). All feature reduction methods demonstrated strong performance, with AUCs ranging from 83.55% to 95.29%. While the ANTI set showed limited performance for classifying SCZ versus HC (AUC = 83.55%), other reduction methods effectively leveraged reward characteristics, yielding significant classification performance (Detailed performance for the stacking models after feature reduction is presented in Table 3 and Supplementary Table S26).

**Table 3.** The performance of stacking models after feature elimination

Feature set	Classification	ACC	SENS	SPEC	AUC (2.5–97.5%percentiles)
MOTI	MDD versus HC	80.88%	70.97%	89.19%	90.50% (78.47%–98.96%)
	SCZ versus HC	79.38%	71.74%	86.27%	88.41% (76.57%–96.12%)
ANTI	MDD versus HC	75.00%	80.65%	70.27%	88.75% (75.44%–97.92%)
	SCZ versus HC	76.29%	76.09%	76.47%	83.55% (70.61%–94.09%)
LOSS	MDD versus HC	83.82%	80.65%	86.49%	93.55% (83.88%–98.96%)
	SCZ versus HC	78.35%	65.22%	90.20%	90.37% (80.95%–97.22%)
HIGH	MDD versus HC	82.35%	83.87%	81.08%	92.24% (82.14%–99.24%)
	SCZ versus HC	78.35%	69.57%	86.27%	88.32% (77.68%–96.70%)
TOP 8 co-emerging	MDD versus HC	83.82%	77.42%	89.19%	95.29% (87.55%–100.00%)
	SCZ versus HC	80.41%	67.39%	92.16%	91.73% (83.04%–98.04%)

*Note:* ACC, ‘accuracy’; SENS, ‘sensitivity’; SPEC, ‘specificity’; AUC, ‘area under the curve’; MOTI, ‘motivation’. See the detailed performance of the stacking model in Supplementary Table S19.

## Discussion

Our study evaluated the performance of ML models in classifying MDD and SCZ from HC. The stacking model consistently achieved superior performance in distinguishing MDD and SCZ from HC, demonstrating strong generalization on independent datasets, although its performance decreased when distinguishing between MDD and SCZ. Feature importance analysis highlighted that the motivation dimension was crucial for distinguishing MDD versus HC and SCZ versus HC from the TEP perspective. Additionally, the FEEL component was more influential in classifying MDD, while the PRED component played a larger role in SCZ versus HC classification. High reward magnitudes had a greater impact than low or control magnitudes, and loss-related consequences were significantly more important than gains. Following feature refinement, the stacking model consistently demonstrated strong classification performance across all comparisons.

Our stacking model demonstrated exceptional performance in classifying both MDD versus HC and SCZ versus HC. Consistent with our results, prior studies have also shown the effectiveness of stacking in distinguishing MDD, achieving AUC from 98.47% to 100.00% (Mahendran et al., 2020). Furthermore, a study leveraging cognitive or socio-cognitive characteristics as classification features for SCZ and bipolar disorder underscored the effectiveness of stacking algorithms in identifying disease-specific impairments (Raio et al., 2023). In our study, the stacking model’s superior performance likely stemmed from its ability to integrate multiple models and classifiers tailored to symptom-representative features, thereby enhancing both accuracy and robustness. However, the classification performance between MDD and SCZ was lower than anticipated, with an AUC of 57.75%, highlighting the difficulty of distinguishing these disorders based on hedonic components. This result underscores the shared characteristics of hedonic processing deficits in MDD and SCZ, aligning with the conceptualization of the positive valence system within the RDoC framework (Hess et al., 2016).

Interestingly, the feature of motivation was identified as the most significant component of hedonic processing in the TEP framework in this study, which may serve as a critical behavioral marker distinguishing MDD and SCZ from healthy controls. Transdiagnostic research highlights that both MDD and SCZ exhibit motivational impairments, particularly in effort-cost computation. However, the underlying mechanisms of motivational dysfunction may differ between MDD and SCZ. In MDD, motivational impairments are primarily driven by deficits in subjective reward valuation and effort-cost estimation, reflecting the interplay between anticipatory pleasure and effort–cost computation (Treadway, Bossaller, Shelton, & Zald, 2012). Conversely, in SCZ, these dysfunctions are largely attributed to disruptions in reward information integration and learning processes, with particularly pronounced impairments in reward prediction (Somlai et al., 2011). Furthermore, research emphasizes the central role of dopamine dysregulation in motivational impairments (Simpson et al., 2022; Wise, 2004). In both MDD and SCZ, motivational dysfunction is associated with disruptions in dopamine pathways, particularly within the mesolimbic and mesocortical circuits. These disruptions may reduce the willingness to exert effort, contributing to the manifestation of apathy symptoms in both disorders (Su & Si, 2022). Thus, our findings underscore the critical role of motivation in understanding anhedonia symptoms across both SCZ and MDD from a transdiagnostic perspective.

For anticipatory pleasure, the FEEL component emerged as more influential in classifying MDD, whereas the PRED component played a greater role in SCZ classification. Anticipatory pleasure plays a crucial role in understanding anhedonia across both MDD and SCZ, though the underlying mechanisms differ between the two disorders. In MDD, evidence indicates diminished reward sensitivity and weakened responses in hedonic processing, which describes a general reduction in emotional reactivity (Alloy, Olino, Freed, & Nusslock, 2016; Proudfit et al., 2015). In contrast, SCZ is marked by reduced sensitivity to reward value, reflecting a rigidity in adapting choices to changing rewards (Martinelli, Rigoli, Dolan, & Shergill, 2018). This inflexibility, negatively correlated with abnormal salience experiences, likely contributes to the decline in differential aspects of anticipatory pleasure (Yaple, Tolomeo, & Yu, 2021). MDD patients often overemphasize negative information, resulting in blunted hedonic processing (Rutledge et al., 2017; Ubl et al., 2015), while SCZ patients frequently struggle to predict rewarding outcomes, impairing reinforcement learning abilities. In the separate classification of MDD and SCZ from HC, both disorders showed impairments in anticipatory pleasure. Although direct classification between SCZ and MDD yielded limited accuracy (AUC = 57.75%), the differential importance of features – particularly ‘prediction’ and ‘feeling’– suggests that the two disorders may involve distinct patterns of reward dysfunction. These findings underscore the need for future studies to incorporate such cognitive dimensions when comparing SCZ and MDD.

Contrary to our hypothesis, we found that reward LOSS features were more critical than reward GAIN features in distinguishing MDD and SCZ. Prior research on MDD has primarily emphasized diminished responsiveness to gains. For example, Liu et al. (2014) demonstrated that individuals with MDD may have difficulty processing gain-related outcomes and exhibit impaired reward-seeking behavior. Our findings align more closely with Bylsma’s (2021) perspective, which emphasizes heightened emotional reactivity and cognitive biases toward losses, leading to amplified negative affect through biased learning processes. This heightened sensitivity to adverse consequences may intensify negative affect, impair feedback adaptation (Berry et al., 2019), and exacerbate anhedonia symptoms in MDD (Li et al., 2022). This finding aligns with the ECI theory in MDD, which suggests blunted emotional responses to both positive and negative outcomes (Bylsma, 2021). However, the prominence of loss-related features in MDD observed here remains unclear and warrants further investigation. In SCZ, impaired loss processing affects the recognition and response to negative outcomes, contributing to cognitive and emotional deficits (Martinelli et al., 2018). Moreover, asymmetric reward learning – characterized by heightened responses to losses and impaired reward updating – also amplifies the influence of loss-related features. Consistent with this evidence, neuroimaging studies have shown increased activation in the ventral striatum and ventromedial prefrontal cortex during loss-avoidance conditions, suggesting a neural bias toward the avoidance of negative outcomes (Waltz et al., 2018). These findings extend beyond gain-processing deficits and underscore the distinct yet overlapping role of loss-related rewards in MDD and SCZ, informing transdiagnostic models of reward dysfunction (Mathews, Chang, Devlin, & Levin, 2023).

This study had several limitations, despite achieving excellent performance by using features of reward processing components. First, the features from the MID task did not include all relevant reward processing components in the TEP framework. The limited sample size constrained our ability to empirically validate the theoretical structure underlying the TEP framework. The lack of clinical subtype data, such as melancholic and atypical depression, limits subtype-specific analyses in MDD and precludes investigation of potential differences in reward processing abnormalities across subtypes. While both MDD and SCZ exhibit reward prediction errors and update action values based on feedback, the MID task does not capture the reinforcement learning process (Frydecka et al., 2021). And the PRED and RECA feature sets were based on self-report measures, whereas the FEEL, CONS, and MOTI were derived from the MID task. We calculated the average feature importance (absolute SHAP values |SHAP|) using corrected paired-sample t-tests across 100 bootstrapped iterations. The results showed that features from the MID task were significantly more influential than those from self-report measures in the classification models (MDD versus HC: *t* = 385.05, *df* = 100, *p* < 0.001; SCZ versus HC: *t* = −256.03, *df* = 100, *p* < 0.001). Additionally, future research could integrate multimodal data, such as neuroimaging, to gain a deeper understanding of how different components of reward processing are affected across disorders. By employing computational modeling, researchers could establish patterns of impaired hedonic processing in different diseases to improve diagnosis and treatment for anhedonia.

## Conclusion

Our study demonstrates that the stacking model achieved superior performance with strong generalization to independent datasets, although its effectiveness in distinguishing between MDD and SCZ was relatively limited. Feature importance analysis revealed that motivation was the most critical dimension for distinguishing both MDD and SCZ. Additionally, the feeling component was more influential in MDD, while the prediction component was more significant for SCZ. High reward magnitudes and loss consequences were found to have a stronger impact in both MDD and SCZ. This transdiagnostic perspective, leveraging multidimensional hedonic processing symptom-related features and the application of the STACKING model, proves to be an effective and reliable approach for understanding reward processing deficits in MDD and SCZ.

## Supporting information

Huang et al. supplementary material Huang et al. supplementary material

## Supplementary material

The supplementary material for this article can be found at http://doi.org/10.1017/S0033291725101207.
