# Supplementary material for: Leveraging stacked classifiers for exploring the role of hedonic processing between major depressive disorder and schizophrenia
Source: Psychol Med. 2025 Jul 23;55:e211. doi: 10.1017/S0033291725101207 (PMC12315667; doi:10.1017/S0033291725101207)
Supplement: Huang et al. supplementary material [file S0033291725101207sup001.docx]

Table of Contents

**[Table S1 Post-hoc analysis of ANOVA results for participants’ socio-demographic information 3](#_Toc1092268127)**

**[Table S2 Definition of abbreviations for each feature set 4](#_Toc1060195933)**

**[Table S3 Classification principles of the models and feature importance algorithms. 6](#_Toc1041226772)**

**[Table S4 Features with Variance Inflation Factor (VIF) > 10 in LR classification 7](#_Toc54117601)**

**[Table S5 Classification performance before and after removing features with VIF > 10. 8](#_Toc1168937326)**

**[Table S6 Comparison of Classification Performance (ACC and AUC) Before and After Removing VIF > 10 Variables in MDD vs. HC and SCZ vs. HC in LR 9](#_Toc1149235326)**

**[Table S7 Performance of binary classification models for MDD vs. HC on training and testing sets. 10](#_Toc730202964)**

**[Table S8 Performance of binary classification models for SCZ vs. HC on training and testing sets. 11](#_Toc1799656990)**

**[Table S9 Binary classification performance of RF and XGB models before and after regularization 12](#_Toc1675346582)**

**[Table S10 The MLP and stacking performance on MDD vs. HC and SCZ vs. HC 13](#_Toc1891907857)**

**[Table S11 Comparison of AUC Performance in MDD vs. HC and SCZ vs. HC Classification Models with Demographic Variables 14](#_Toc1652475117)**

**[Table S12 Classification Performance for Subgroups Stratified by Symptom Severity 15](#_Toc1890768415)**

**[Table S13 Classification Performance for Subgroups Stratified by Symptom Severity and Subgroups Stratified by Medication Status 16](#_Toc1829226246)**

**[Table S14 Comparison of feature importance in hedonic components for MDD vs. HC. 17](#_Toc429626070)**

**[Table S15 Comparison of feature importance in reward consequences for MDD vs. HC. 18](#_Toc885337276)**

**[Table S16 Comparison of feature importance in reward magnitudes for MDD vs. HC. 19](#_Toc2096891316)**

**[Table S17 Comparison of feature importance in reward consequences * magnitudes for MDD vs. HC. 20](#_Toc98217095)**

**[Table S18 Comparison of feature importance in hedonic components for SCZ vs. HC. 21](#_Toc1467274769)**

**[Table S19 Comparison of feature importance in reward consequences for SCZ vs. HC 22](#_Toc932324082)**

**[Table S20 Comparison of feature importance in reward magnitudes for SCZ vs. HC. 23](#_Toc1530157662)**

**[Table S21 Comparison of feature importance in reward consequences * magnitudes for SCZ vs. HC. 24](#_Toc1243152409)**

**[Table S22 Comparison of feature set importance between MDD vs. HC and SCZ vs. HC 25](#_Toc794136400)**

**[Table S23 Feature set importance across additional algorithms for MDD vs. HC 26](#_Toc439608695)**

**[Table S24 Feature set importance across additional algorithms for SCZ vs. HC. 27](#_Toc1159591185)**

**[Table S25 Top 20 features ranked by importance for MDD vs. HC and SCZ vs. HC using stacking models 28](#_Toc834949770)**

**[Table S26 Detailed performance of stacking models after feature elimination. 29](#_Toc1342634892)**

**[Figure S1 the ACC and AUC Learning Curve of the RF for MDD vs. HC and SCZ vs. HC 30](#_Toc2053950815)**

**[Figure S2 the ACC and AUC Learning Curve of the XGB for MDD vs. HC and SCZ vs. HC 31](#_Toc2099205827)**

**[Figure S3 Figure Confusion Matrix of MDD vs. SCZ 32](#_Toc343497826)**

**[Figure S4 SHAP Plot for MOTI in MDD and SCZ 33](#_Toc731918446)**

**[Figure S5 SHAP Plot for FEEL in MDD and SCZ 34](#_Toc566991906)**

**[Figure S6 SHAP Plot for PRED in MDD and SCZ 35](#_Toc1048022403)**

**[Figure S7 SHAP Plot for CONS in MDD and SCZ 36](#_Toc451654527)**

**[Figure S8 SHAP Plot for RECA in MDD and SCZ 37](#_Toc1750426791)**

**[Note: The implementation and hyperparameter configurations of MLP and TabNet. 38](#_Toc1044596084)**

**Table S1** **Post-hoc analysis of ANOVA results for participants’ socio-demographic information**

|  | (I)Group | (J)Group | 95% confidence interval | | p |
| --- | --- | --- | --- | --- | --- |
|  |  |  | lower limit | upper limit |  |
| Gender | HC | MDD | - | - | 0.72 |
|  | HC | SCZ | - | - | 0.016 |
|  | MDD | SCZ | - |  | 0.002 |
| Age | HC | MDD | -7.68 | -3.14 | <.001 |
|  | HC | SCZ | -5.36 | -0.8 | 0.005 |
|  | MDD | SCZ | -0.01 | 4.67 | 0.052 |
| Education | HC | MDD | 0.251 | 2.149 | 0.009 |
|  | HC | SCZ | -1.365 | 0.443 | 0.454 |
|  | MDD | SCZ | -2.636 | -0.685 | <.001 |

Note: MDD: Major Depressive Disorder; SCZ: Schizophrenia; HC: Healthy Controls.

**Table S2 Definition of abbreviations for each feature set**

| Feature Category | Component Label | Feature Name |
| --- | --- | --- |
| Reward Components (N=114) | MOTI  (N=6) | MOTI_RT_GH, MOTI_RT_GL, MOTI_RT_GC, MOTI_RT_LH, MOTI_RT_LL, MOTI_RT_LC. |
|  | FEEL  (N=12) | FEEL_AV_GH, FEEL_AV_GL, FEEL_AV_GC, FEEL_AV_LH, FEEL_AV_LL, FEEL_AV_LC, FEEL_AA_GH, FEEL_AA_GL, FEEL_AA_GC, FEEL_AA_LH, FEEL_AA_LL, FEEL_AA_LC. |
|  | PRED  (N=36) | PRED_AV_GH, PRED_AV_GL, PRED_AV_GC, PRED_AV_LH, PRED_AV_LL, PRED_AV_LC, PRED_AA_GH, PRED_AA_GL, PRED_AA_GC, PRED_AA_LH, PRED_AA_LL, PRED_AA_LC, PRED_CV_GH, PRED_CV_GL, PRED_CV_GC, PRED_CV_LH, PRED_CV_LL, PRED_CV_LC, PRED_CA_GH, PRED_CA_GL, PRED_CA_GC, PRED_CA_LH, PRED_CA_LL, PRED_CA_LC, PRED_CA_NLH, PRED_CA_NLL, PRED_CA_NLC, PRED_CV_NGH, PRED_CV_NGL, PRED_CV_NGC, PRED_CV_NLH, PRED_CV_NLL, PRED_CV_NLC, PRED_CA_NGH, PRED_CA_NGL, PRED_CA_NGC. |
|  | CONS  (N=24) | CONS_CV_GH, CONS_CV_GL, CONS_CV_GC, CONS_CV_LH, CONS_CV_LL, CONS_CV_LC, CONS_CA_GH, CONS_CA_GL, CONS_CA_GC, CONS_CA_LH, CONS_CA_LL, CONS_CA_LC, CONS_CA_NGH, CONS_CA_NGL, CONS_CA_NGC, CONS_CA_NLH, CONS_CA_NLL, CONS_CA_NLC, CONS_CV_NGH, CONS_CV_NGL, CONS_CV_NGC, CONS_CV_NLH, CONS_CV_NLL, CONS_CV_NLC. |
|  | RECA  (N=36) | RECA_AV_GH, RECA_AV_GL, RECA_AV_GC, RECA_AV_LH, RECA_AV_LL, RECA_AV_LC, RECA_AA_GH, RECA_AA_GL, RECA_AA_GC, RECA_AA_LH, RECA_AA_LL, RECA_AA_LC, RECA_CV_GH, RECA_CV_GL, RECA_CV_GC, RECA_CV_LH, RECA_CV_LL, RECA_CV_LC, RECA_CA_GH, RECA_CA_GL, RECA_CA_GC, RECA_CA_LH, RECA_CA_LL, RECA_CA_LC, RECA_CA_NGH, RECA_CA_NGL, RECA_CA_NGC, RECA_CA_NLH, RECA_CA_NLL, RECA_CA_NLC, RECA_CV_NGH, RECA_CV_NGL, RECA_CV_NGC, RECA_CV_NLH, RECA_CV_NLL, RECA_CV_NLC. |
| Reward Consequences (N=114) | GAIN (N=57) | MOTI_RT_GH, MOTI_RT_GL, MOTI_RT_GC, FEEL_AV_GH, FEEL_AV_GL, FEEL_AV_GC, FEEL_AA_GH, FEEL_AA_GL, FEEL_AA_GC, PRED_AV_GH, PRED_AV_GL, PRED_AV_GC, PRED_AA_GH, PRED_AA_GL, PRED_AA_GC, PRED_CV_GH, PRED_CV_GL, PRED_CV_GC, PRED_CA_GH, PRED_CA_GL, PRED_CA_GC, PRED_CV_NGH, PRED_CV_NGL, PRED_CV_NGC, PRED_CA_NGH, PRED_CA_NGL, PRED_CA_NGC, CONS_CV_GH, CONS_CV_GL, CONS_CV_GC, CONS_CA_GH, CONS_CA_GL, CONS_CA_GC, CONS_CA_NGH, CONS_CA_NGL, CONS_CA_NGC, CONS_CV_NGH, CONS_CV_NGL, CONS_CV_NGC, RECA_AV_GH, RECA_AV_GL, RECA_AV_GC, RECA_AA_GH, RECA_AA_GL, RECA_AA_GC, RECA_CV_GH, RECA_CV_GL, RECA_CV_GC, RECA_CA_GH, RECA_CA_GL, RECA_CA_GC, RECA_CA_NGH, RECA_CA_NGL, RECA_CA_NGC, RECA_CV_NGH, RECA_CV_NGL, RECA_CV_NGC. |
|  | LOSS  (N=57) | MOTI_RT_LH, MOTI_RT_LL, MOTI_RT_LC, FEEL_AV_LH, FEEL_AV_LL, FEEL_AV_LC, FEEL_AA_LH, FEEL_AA_LL, FEEL_AA_LC, PRED_AV_LH, PRED_AV_LL, PRED_AV_LC, PRED_AA_LH, PRED_AA_LL, PRED_AA_LC, PRED_CV_LH, PRED_CV_LL, PRED_CV_LC, PRED_CA_LH, PRED_CA_LL, PRED_CA_LC, PRED_CA_NLH, PRED_CA_NLL, PRED_CA_NLC, PRED_CV_NLH, PRED_CV_NLL, PRED_CV_NLC, CONS_CV_LH, CONS_CV_LL, CONS_CV_LC, CONS_CA_LH, CONS_CA_LL, CONS_CA_LC, CONS_CA_NLH, CONS_CA_NLL, CONS_CA_NLC, CONS_CV_NLH, CONS_CV_NLL, CONS_CV_NLC, RECA_AV_LH, RECA_AV_LL, RECA_AV_LC, RECA_AA_LH, RECA_AA_LL, RECA_AA_LC, RECA_CV_LH, RECA_CV_LL, RECA_CV_LC, RECA_CA_LH, RECA_CA_LL, RECA_CA_LC, RECA_CA_NLH, RECA_CA_NLL, RECA_CA_NLC, RECA_CV_NLH, RECA_CV_NLL, RECA_CV_NLC. |
|  |  |  |
|  |  |  |
| Reward Magnitudes (N=114) | GH (N=19) | MOTI_RT_GH, FEEL_AV_GH, FEEL_AA_GH, PRED_AV_GH, PRED_AA_GH, PRED_CV_GH, PRED_CA_GH, PRED_CV_NGH, PRED_CA_NGH, CONS_CV_GH, CONS_CA_GH, CONS_CA_NGH, CONS_CV_NGH, RECA_AV_GH, RECA_AA_GH, RECA_CV_GH, RECA_CA_GH, RECA_CA_NGH, RECA_CV_NGH. |
|  | GL (N=19) | MOTI_RT_GL, FEEL_AV_GL, FEEL_AA_GL, PRED_AV_GL, PRED_AA_GL, PRED_CV_GL, PRED_CA_GL, PRED_CV_NGL, PRED_CA_NGL, CONS_CV_GL, CONS_CA_GL, CONS_CA_NGL, CONS_CV_NGL, RECA_AV_GL, RECA_AA_GL, RECA_CV_GL, RECA_CA_GL, RECA_CA_NGL, RECA_CV_NGL. |
|  |  |  |
|  | GN (N=19) | MOTI_RT_GC, FEEL_AV_GC, FEEL_AA_GC, PRED_AV_GC, PRED_AA_GC, PRED_CV_GC, PRED_CA_GC, PRED_CV_NGC, PRED_CA_NGC, CONS_CV_GC, CONS_CA_GC, CONS_CA_NGC, CONS_CV_NGC, RECA_AV_GC, RECA_AA_GC, RECA_CV_GC, RECA_CA_GC, RECA_CA_NGC, RECA_CV_NGC. |
|  |  |  |
|  | LH (N=19) | MOTI_RT_LH, FEEL_AV_LH, FEEL_AA_LH, PRED_AV_LH, PRED_AA_LH, PRED_CV_LH, PRED_CA_LH, PRED_CA_NLH, PRED_CV_NLH, CONS_CV_LH, CONS_CA_LH, CONS_CA_NLH, CONS_CV_NLH, RECA_AV_LH, RECA_AA_LH, RECA_CV_LH, RECA_CA_LH, RECA_CA_NLH, RECA_CV_NLH. |
|  |  |  |
|  | LL (N=19) | MOTI_RT_LL, FEEL_AV_LL, FEEL_AA_LL, PRED_AV_LL, PRED_AA_LL, PRED_CV_LL, PRED_CA_LL, PRED_CA_NLL, PRED_CV_NLL, CONS_CV_LL, CONS_CA_LL, CONS_CA_NLL, CONS_CV_NLL, RECA_AV_LL, RECA_AA_LL, RECA_CV_LL, RECA_CA_LL, RECA_CA_NLL, RECA_CV_NLL. |
|  |  |  |
|  | LN (N=19) | MOTI_RT_LC, FEEL_AV_LC, FEEL_AA_LC, PRED_AV_LC, PRED_AA_LC, PRED_CV_LC, PRED_CA_LC, PRED_CA_NLC, PRED_CV_NLC, CONS_CV_LC, CONS_CA_LC, CONS_CA_NLC, CONS_CV_NLC, RECA_AV_LC, RECA_AA_LC, RECA_CV_LC, RECA_CA_LC, RECA_CA_NLC, RECA_CV_NLC. |
|  |  |  |

Note: MOTI: Motivation; FEEL: Feeling; PRED: Prediction; CONS: Consummatory pleasure; RECA: Remembered pleasure; GAIN: Gain Reward; LOSS: Avoid Loss Reward; RT: Reaction Time; Meaning of the first letter of the acronym after the first “_” in Feature name: A: Anticipatory pleasure; C: Consummatory Pleasure; Meaning of the second letter of the acronym after the first “_” in Feature name: A: Arousal; V: Valence; GH: Gaining High Reward; GL: Gaining Low Reward; GC: Gaining Control (Zero Rewards); LH: Losing High Reward; LL: Losing Low Reward; LC: Losing Control (Zero Rewards); NGH: No Gaining High (Reward); NGL: No Gaining Low (Reward); NGC: No Gaining Control (Zero Reward); NLH: No Losing High (Reward); NLL: No Losing Low (Reward); NLC: No Losing Control (Zero Reward).

**Table S3 Classification principles of the models and feature importance algorithms.**

| Classification Principle | Model | Feature importance Algorithm |
| --- | --- | --- |
| Linear Model | Logistic Regression (LR) | - |
| Tree Model | Decision Tree (DT) | GINI |
| Margin-based Model | Support Vector Machine (SVM) | Permutation |
| Instance-based Model | K-Nearest Neighbors (KNN) | - |
| Bayesian Model | Naive Bayes (NB) | - |
| Integrated Model | Random Forest (RF) | GINI |
|  | Gradient Boosting Trees (GBT) | Feature Coverage Gain |
|  | Extreme Gradient Boosting (XGB) | Feature Coverage Gain |
|  | Stacking (STACKING) | SHAP |

Note: Selected models with diverse classification principles to ensure a robust learning process.

Linear Model: Logistic Regression (LR) - Assumes a linear relationship between features and the target class.

Tree Model: Decision Tree (DT) - Makes decisions based on a sequence of binary splits, capturing nonlinear relationships.

Instance-based Model: K-Nearest Neighbors (KNN) - Classifies based on the majority class among the closest training examples.

Bayesian Model: Naive Bayes (NB) - Applies Bayes' theorem with an assumption of independence between features.

Margin-based Model: Support Vector Machine (SVM) - Finds the hyperplane that best separates classes by maximizing the margin between them.

Integrated Model: Random Forest (RF) - Combines multiple decision trees to improve classification accuracy by averaging predictions.

Gradient-based Ensemble Models: Gradient Boosting Trees (GBT), Extreme Gradient Boosting (XGB) - Build sequential trees where each tree corrects errors made by the previous one, improving prediction accuracy over iterations.

**Table S4** **Features with Variance Inflation Factor (VIF) > 10 in LR classification**

|  | MDD vs. HC | | SCZ vs. HC | |
| --- | --- | --- | --- | --- |
| rank | Feature name | VIF | Feature name | VIF |
| 1 | FEEL_AA_GH | 22.2305 | CONS_CA_GH | 17.7774 |
| 2 | RECA_CA_GC | 16.5401 | FEEL_AA_GH | 17.0690 |
| 3 | RECA_CA_GH | 15.3321 | RECA_CA_GC | 16.0097 |
| 4 | CONS_CA_GH | 14.4383 | RECA_AA_GC | 15.6886 |
| 5 | RECA_CV_GH | 14.3347 | FEEL_AA_GC | 14.2346 |
| 6 | RECA_AA_GH | 14.2187 | RECA_CA_NGC | 14.0293 |
| 7 | RECA_CA_NGC | 14.0729 | RECA_CA_LC | 13.4612 |
| 8 | PRED_AA_GC | 13.5170 | FEEL_AA_LC | 12.6755 |
| 9 | RECA_CA_LC | 13.4930 | RECA_CA_GH | 12.5212 |
| 10 | RECA_AA_GC | 13.3419 | FEEL_AA_LH | 11.6630 |
| 11 | FEEL_AV_GH | 13.3268 | RECA_CV_LH | 11.6253 |
| 12 | FEEL_AA_GC | 12.7489 | FEEL_AV_LH | 10.7987 |
| 13 | FEEL_AA_LH | 11.9466 | FEEL_AA_LL | 10.6558 |
| 14 | PRED_CA_GC | 11.8624 | PRED_CV_LH | 10.6387 |
| 15 | FEEL_AA_GL | 11.8170 | RECA_CA_NLL | 10.5100 |
| 16 | RECA_CA_NLC | 11.7504 | RECA_AA_GH | 10.4152 |
| 17 | FEEL_AV_LL | 11.6482 | RECA_CV_GH | 10.1624 |
| 18 | CONS_CV_GH | 11.3750 | RECA_AA_LC | 10.1612 |
| 19 | PRED_CV_GH | 10.3903 | PRED_CA_GH | 10.1447 |
| 20 | RECA_CA_LH | 10.2676 | RECA_CA_NGH | 10.1195 |
| 21 | FEEL_AA_LC | 10.1863 | RECA_AA_GL | 10.0399 |
| 22 | RECA_AA_LL | 10.0363 |  |  |

Note: MDD: Major Depressive Disorder; SCZ: Schizophrenia; HC: Healthy Controls; FEEL: Feeling; RECA: Remembered pleasure; CONS: Consummatory pleasure; PRED: Prediction; Meaning of the second letter of the acronym after the first “_” in Feature name: A-Arousal; V-Valence; NGH=No Gaining High (Reward); NGL-No Gaining Low (Reward); NGC- No Gaining Control (Zero reward); NLH: No Losing High (Reward); NLL: No Losing Low (Reward); NLC: No Losing Control (Zero Reward).

**Table S5 Classification performance before and after removing features with VIF > 10.**

|  | Feature Number (N) | ACC | SENS | SPEC | AUC |
| --- | --- | --- | --- | --- | --- |
| MDD vs. HC | N=114 | 93.02% | 92.86% | 93.10% | 95.57% |
|  | N=92 | 90.69% | 85.71% | 93.10% | 96.55% |
| SCZ vs. HC | N=114 | 90.70% | 84.21% | 95.83% | 96.49% |
|  | N=93 | 95.35% | 94.74% | 95.83% | 96.71% |

Note: MDD: Major Depressive Disorder; SCZ: Schizophrenia; HC: Healthy Controls; ACC: accuracy; SENS: sensitivity; SPEC: specificity; AUC: Area Under the Receiver Operating Characteristic Curve.

**Table S6 Comparison of Classification Performance (ACC and AUC) Before and After Removing VIF > 10 Variables in MDD vs. HC and SCZ vs. HC in LR**

| Classification | Varviables | All Features  (mean, sd) | | VIF<10 Features  (mean, sd) | | t | df | Cohen's d | p |
| --- | --- | --- | --- | --- | --- | --- | --- | --- | --- |
| MDD vs. HC | ACC | 0.8751 | 0.0478 | 0.8788 | 0.0478 | -1.7074 | 1998.0000 | -0.0764 | 0.3516 |
|  | AUC | 0.9486 | 0.0294 | 0.9504 | 0.0292 | -1.3482 | 1997.9356 | -0.0603 | 0.7110 |
| SCZ vs. HC | ACC | 0.8749 | 0.0476 | 0.8736 | 0.0495 | 0.6106 | 1994.9112 | 0.0273 | 1.0000 |
|  | AUC | 0.9505 | 0.0282 | 0.9492 | 0.0306 | 1.0020 | 1985.2086 | 0.0448 | 1.0000 |

Note: MDD: Major Depressive Disorder; SCZ: Schizophrenia; HC: Healthy Controls; ACC: accuracy; AUC: Area Under the Receiver Operating Characteristic Curve; LR: Logistic Regression.

To robustly evaluate the performance of LR models before and after removing variables with VIF > 10, we conducted 1000 iterations of model training and testing for both MDD vs. HC and SCZ vs. HC classifications using randomly shuffled data. In each iteration, ACC and AUC values were computed and recorded. Independent samples t-tests were conducted to compare the classification performance (ACC and AUC) between the two models in both binary classification tasks. Bonferroni-corrected p-values are reported.

**Table S7 Performance of binary classification models for MDD vs. HC on training and testing sets.**

| MODEL |  | ACC | SENS | SPEC | AUC |
| --- | --- | --- | --- | --- | --- |
| STACKING | training set | 83.43% | 88.24% | 78.57% | 100.00% |
|  | testing set | 88.37% | 100.00% | 82.76% | 97.04% |
| XGB | training set | 86.39% | 87.06% | 85.71% | 100.00% |
|  | testing set | 90.70% | 85.71% | 93.10% | 96.31% |
| RF | training set | 84.62% | 88.24% | 80.95% | 98.95% |
|  | testing set | 88.37% | 92.86% | 86.21% | 95.81% |
| LR | training set | 86.98% | 85.88% | 88.10% | 99.94% |
|  | testing set | 93.02% | 92.86% | 93.10% | 95.57% |
| SVM | training set | 85.21% | 80.49% | 89.66% | 100.00% |
|  | testing set | 88.37% | 88.24% | 88.46% | 95.70% |
| GBT | training set | 81.66% | 83.53% | 79.76% | 99.87% |
|  | testing set | 88.37% | 85.71% | 89.66% | 94.58% |
| KNN | training set | 84.62% | 84.71% | 84.52% | 97.31% |
|  | testing set | 86.05% | 85.71% | 86.21% | 93.60% |
| NB | training set | 82.84% | 87.06% | 78.57% | 93.50% |
|  | testing set | 81.40% | 85.71% | 79.31% | 93.10% |
| DT | training set | 78.11% | 78.10% | 78.11% | 98.77% |
|  | testing set | 86.05% | 85.71% | 86.21% | 84.98% |

Note：Shown here is the binary classification of all features in MDD vs. HC. MDD: Major Depressive Disorder; SCZ: Schizophrenia; HC: Healthy Controls; ACC: accuracy; SENS: sensitivity; SPEC: specificity; AUC: Area Under the Receiver Operating Characteristic Curve; STACKING: Stacking Model; XGB: eXtreme Gradient Boosting; RF: Random Forest; LR: Logistic Regression; SVM: Support Vector Machine; GBT: Gradient Boosting Tree; KNN: k-Nearest Neighbors; NB: Naive Bayes; DT: Decision Tree.

**Table S8 Performance of binary classification models for SCZ vs. HC on training and testing sets.**

| MODEL |  | ACC | SENS | SPEC | AUC |
| --- | --- | --- | --- | --- | --- |
| STACKING | training set | 87.65% | 85.19% | 89.89% | 100.00% |
|  | testing set | 88.37% | 94.74% | 79.17% | 96.05% |
| XGB | training set | 85.88% | 82.72% | 88.76% | 100.00% |
|  | testing set | 86.05% | 97.47% | 79.17% | 94.74% |
| RF | training set | 88.24% | 86.42% | 89.89% | 100.00% |
|  | testing set | 93.02% | 94.74% | 91.67% | 94.96% |
| LR | training set | 90.70% | 84.21% | 95.83% | 100.00% |
|  | testing set | 90.70% | 84.21% | 95.83% | 96.49% |
| SVM | training set | 89.41% | 85.19% | 93.26% | 100.00% |
|  | testing set | 88.37% | 68.42% | 91.67% | 94.30% |
| GBT | training set | 81.76% | 75.31% | 87.64% | 100.00% |
|  | testing set | 83.72% | 89.47% | 79.17% | 92.11% |
| KNN | training set | 81.76% | 69.14% | 93.26% | 96.17% |
|  | testing set | 86.05% | 85.71% | 86.21% | 93.60% |
| NB | training set | 81.76% | 75.13% | 87.64% | 93.97% |
|  | testing set | 86.05% | 89.47% | 83.33% | 85.86% |
| DT | training set | 81.18% | 79.10% | 83.16% | 99.94% |
|  | testing set | 81.40% | 84.21% | 79.17% | 85.86% |

Note：Shown here is the binary classification of all features in SCZ vs. HC. SCZ: Schizophrenia; HC: Healthy Controls; ACC: accuracy; SENS: sensitivity; SPEC: specificity; AUC: Area Under the Receiver Operating Characteristic Curve; STACKING: Stacking Model; XGB: eXtreme Gradient Boosting; RF: Random Forest; LR: Logistic Regression; SVM: Support Vector Machine; GBT: Gradient Boosting Tree; KNN: k-Nearest Neighbors; NB: Naive Bayes; DT: Decision Tree.

**Table S9 Binary classification performance of RF and XGB models before and after regularization**

| MODEL | Classification |  | ACC | SENS | SPEC | AUC |
| --- | --- | --- | --- | --- | --- | --- |
| RF | MDD vs. HC | training set | 84.62% | 88.24% | 80.95% | 98.95% |
|  | MDD vs. HC | testing set | 88.37% | 92.86% | 86.21% | 95.81% |
| Regularized RF | MDD vs. HC | training set | 84.02% | 87.06% | 80.95% | 98.60% |
|  | MDD vs. HC | testing set | 88.37% | 92.86% | 86.21% | 95.81% (89.23%-100.00%) |
| RF | SCZ vs. HC | training set | 88.24% | 86.42% | 89.89% | 100.00% |
|  | SCZ vs. HC | testing set | 93.02% | 94.74% | 91.67% | 94.96% |
| Regularized RF | SCZ vs. HC | training set | 90.00% | 88.89% | 91.01% | 99.15% |
|  | SCZ vs. HC | testing set | 93.02% | 94.74% | 91.67% | 95.39% (88.00%-100.00%) |
| XGB | MDD vs. HC | training set | 86.39% | 87.06% | 85.71% | 100.00% |
|  | MDD vs. HC | testing set | 90.70% | 85.71% | 93.10% | 96.31% |
| Regularized XGB | MDD vs. HC | training set | 86.98% | 89.41% | 84.52% | 100% |
|  | MDD vs. HC | testing set | 93.02% | 92.86% | 93.10% | 97.04% (90.97%-100%) |
| XGB | SCZ vs. HC | training set | 85.88% | 82.72% | 88.76% | 100.00% |
|  | SCZ vs. HC | testing set | 86.05% | 97.47% | 79.17% | 94.74% |
| Regularized XGB | SCZ vs. HC | training set | 88.24% | 87.65% | 88.76% | 100% |
|  | SCZ vs. HC | testing set | 90.70% | 94.74% | 87.50% | 95.18% (87.23%-100.00%) |

Note: MDD: Major Depressive Disorder; SCZ: Schizophrenia; HC: Healthy Controls; ACC: accuracy; SENS: sensitivity; SPEC: specificity; AUC: Area Under the Receiver Operating Characteristic Curve; RF: Random Forest; XGB: eXtreme Gradient Boosting.

RF mitigates overfitting by tuning hyperparameters like max_depth, min_samples_split, and min_samples_leaf. XGB incorporates regularization via L1 (reg_alpha), promoting sparsity by penalizing coefficient magnitudes, and L2 (reg_lambda), reducing complexity and preventing overfitting.

**Table S10 The MLP and stacking performance on MDD vs. HC and SCZ vs. HC**

| Model | Classification |  | ACC | SENS | SPEC | AUC |
| --- | --- | --- | --- | --- | --- | --- |
| TabNet | MDD vs. HC | training set | 78.38% | 80.32% | 76.69% | 86.98% |
|  | MDD vs. HC | testing set | 81.72% | 84.17% | 79.58% | 91.93% (83.04%-98.48%) |
| MLP | MDD vs. HC | training set | 92.07% | 94.34% | 90.09% | 97.78% |
|  |  | testing set | 85.14% | 87.36% | 83.21% | 93.65% (87.32%-99.97%) |
| Stacking | MDD vs. HC | training set | 83.43% | 88.24% | 78.57% | 100.00% |
|  |  | testing set | 88.37% | 100.00% | 82.76% | 97.04% (91.48%-100.00%) |
| TabNet | SCZ vs. HC | training set | 77.62% | 72.85% | 81.86% | 85.81% |
|  | SCZ vs. HC | testing set | 80.66% | 76.30% | 84.46% | 90.74% (80.22%-98.70%) |
| MLP | SCZ vs. HC | training set | 92.57% | 93.73% | 91.55% | 97.72% |
|  |  | testing set | 85.64% | 86.89% | 84.56% | 93.26% (85.86%-100%) |
| Stacking | SCZ vs. HC | training set | 87.65% | 85.19% | 89.89% | 100.00% |
|  |  | testing set | 88.37% | 94.74% | 79.17% | 96.05% (89.29%-100.00%) |

Note: MDD: Major Depressive Disorder; SCZ: Schizophrenia; HC: Healthy Controls; ACC: accuracy; SENS: sensitivity; SPEC: specificity; AUC: Area Under the Receiver Operating Characteristic Curve; MLP: multilayer perceptron.

**Table S11 Comparison of AUC Performance in MDD vs. HC and SCZ vs. HC Classification Models with Demographic Variables**

| Variables | AUC | | t | df | Cohen's d | *p* |
| --- | --- | --- | --- | --- | --- | --- |
|  | Mean | se |  |  |  |  |
| MDD vs. HC - AUC (Reward only) | 0.9717 | 0.0232 | - | - | - | *-* |
| MDD vs. HC - AUC (Reward + EDU) | 0.9642 | 0.0009 | 6.7300 | 1998 | 0.3010 | *0.0000* |
| MDD vs. HC - AUC (Reward + Gender) | 0.9565 | 0.0009 | 13.1414 | 1998 | 0.5877 | *0.0000* |
| MDD vs. HC - AUC (Reward + Age) | 0.9717 | 0.0007 | 0.0000 | 1998 | 0.0000 | *1.0000* |
| SCZ vs. HC - AUC (Reward only) | 0.9626 | 0.0295 | - | - | - | *-* |
| SCZ vs. HC - AUC (Reward + EDU) | 0.9626 | 0.0009 | 0.0000 | 1998 | 0.0000 | *1.0000* |
| SCZ vs. HC - AUC (Reward + Gender) | 0.9649 | 0.0009 | -1.7047 | 1998 | -0.0762 | *0.2652* |
| SCZ vs. HC - AUC (Reward + Age) | 0.9606 | 0.0010 | 1.5193 | 1998 | 0.0679 | *0.3866* |

Note: MDD: Major Depressive Disorder; SCZ: Schizophrenia; HC: Healthy Controls; AUC: Area Under the Receiver Operating Characteristic Curve; EDU: Educational years.

**Table S12 Classification Performance for Subgroups Stratified by Symptom Severity**

| Classification |  | ACC | SENS | SPEC | AUC |
| --- | --- | --- | --- | --- | --- |
| HC vs. MDD_LowBDI | training set | 90.84% | 87.18% | 92.39% | 100.00% |
| (N: 113 vs. 51) | testing set | 81.82% | 66.67% | 90.48% | 93.25%  (83.50% - 99.54%) |
| HC vs. MDD_HighBDI | training set | 89.84% | 79.49% | 94.38% | 99.97% |
| (N: 113 vs. 48) | testing set | 78.79% | 77.78% | 79.17% | 91.67%  (79.29% - 100%) |
| MDD_LowBDI vs. MDD_HighBDI | training set | 63.29% | 67.50% | 58.97% | 100.00% |
| (N: 51 vs. 48) | testing set | 55.00% | 27.27% | 88.89% | 63.64%  (35.42% - 86.81%) |
| HC vs. SCZ_LowNEG | training set | 97.39% | 65.52% | 94.44% | 100.00% |
| (N: 113 vs. 36) | testing set | 86.67% | 71.43% | 91.30% | 93.17% (80.68%-100.00%) |
| HC vs. SCZ_HighNEG | training set | 88.52% | 66.67% | 96.63% | 100.00% |
| (N: 113 vs. 40) | testing set | 93.55% | 85.71% | 95.83% | 99.40% (96.43%-100.00%) |
| SCZ_LowNEG vs. SCZ_HighNEG | training set | 41.67% | 56.25% | 25.00% | 100.00% |
| (N: 36 vs. 40) | testing set | 50.00% | 100.00% | 0.00% | 50.00% (18.18%-81.25%) |

Note: MDD: Major Depressive Disorder; SCZ: Schizophrenia; HC: Healthy Controls; ACC: accuracy; SENS: sensitivity; SPEC: specificity; AUC: area under the curve; MDD_LowBDI: MDD with BDI scores ≤ 16; MDD_HighBDI: MDD with BDI scores > 16; SCZ_LowNEG: SCZ with PANSS Negative subscale scores below the median (≤11); SCZ_HighNEG: SCZ with PANSS Negative subscale scores above the median (> 11).

**Table S13 Classification Performance for Subgroups Stratified by Symptom Severity and Subgroups Stratified by Medication Status**

| Classification |  | ACC | SENS | SPEC | AUC |
| --- | --- | --- | --- | --- | --- |
| HC vs. MDD_Unmed | training set | 90.00% | 65.52% | 97.80% | 100% |
| (N: 113 vs. 37) | testing set | 86.67% | 100.00% | 81.81% | 97.16% (90.00%-100.00%) |
| HC vs. MDD_Med | training set | 90.00% | 86.54% | 92.05% | 100% |
| (N: 113 vs. 62) | testing set | 85.71% | 80.00% | 88.00% | 94.80% (84.40%-100.00%) |
| MDD_Unmed vs. MDD_Med | training set | 58.23% | 91.67% | 6.45% | 100% |
| (N: 37 vs. 62) | testing set | 70.00% | 100% | 0.00% | 72.62%  (46.88%-93.75%) |
| HC vs. SCZ_Lowmed | training set | 90.58% | 86.27% | 93.10% | 100% |
| (N: 113 vs. 60) | testing set | 91.43% | 88.89% | 92.31% | 91.45% (76.61%-100%) |
| HC vs. SCZ_Highmed | training set | 88.79% | 72.41% | 94.25% | 100% |
| (N: 113 vs. 32) | testing set | 89.66% | 66.67% | 92.31% | 73.08% (17.86%-100%) |
| SCZ_Lowmed vs. SCZ_Highmed | training set | 65.75% | 4.17% | 95.92% | 50.00% |
| (N: 60 vs. 32) | testing set | 57.89% | 0.00% | 100.00% | 50.00% (50.00%-50.00%) |

Note: MDD: Major Depressive Disorder; SCZ: Schizophrenia; HC: Healthy Controls; ACC: accuracy; SENS: sensitivity; SPEC: specificity; AUC: area under the curve; MDD_Unmed: MDD who were not currently taking medication; MDD_Med: MDD who were currently taking medication; SCZ_Lowmed: SCZ with a daily antipsychotic dosage ≤ 400 mg CPZ equivalents; SCZ_Highmed: SCZ with a daily antipsychotic dosage > 400 mg CPZ equivalents.

**Table S14 Comparison of feature importance in hedonic components for MDD vs. HC.**

| Hedonic  Components | Feature Importance | (I)Feature set | (J)Feature set | 95% confidence interval | | HSD |
| --- | --- | --- | --- | --- | --- | --- |
|  | (M±SD) |  |  | lower limit | upper limit | p |
| MOTI | 0.1347 (0.0077) | MOTI | FEEL | 0.0848 | 0.0877 | <.001 |
| FEEL | 0.0485(0.0023) | MOTI | PRED | 0.1193 | 0.1222 | <.001 |
| PRED | 0.0140 (0.0005) | MOTI | CONS | 0.0885 | 0.0914 | <.001 |
| CONS | 0.0448(0.0019) | MOTI | RECA | 0.1199 | 0.1228 | <.001 |
| RECA | 0.0134 (00005) | FEEL | PRED | 0.0331 | 0.03592 | <.001 |
|  |  | FEEL | CONS | 0.0022 | 0.0051 | <.001 |
|  |  | FEEL | RECA | 0.0337 | 0.0365 | <.001 |
|  |  | PRED | CONS | -0.0322 | -0.0294 | <.001 |
|  |  | PRED | RECA | -0.0008 | 0.0021 | 0.756 |
|  |  | CONS | RECA | 0.0304 | 0.0325 | <.001 |

Note: Hedonic Components: F (4, 490)=17954.088, p<.001, $\eta_{p}^{2}$=.993; MDD: Major Depressive Disorder; HC: Healthy Controls; HSD: Tukey's Honest Significant Difference test; FEEL: Feeling; PRED: Prediction; CONS: Consummatory pleasure; RECA: Remembered pleasure; MOTI: Motivation.

**Table S15 Comparison of feature importance in reward consequences for MDD vs. HC.**

| Reward  Consequences | Feature Importance | Cohen d 95% confidence interval | | t | p |
| --- | --- | --- | --- | --- | --- |
|  | (M±SD) | lower limit | upper limit |  |  |
| GAIN | 0.0280 (0.0008) | -6.192 | -4.968 | -39.664 | <.001 |
| LOSS | 0.3262 (0.0009) |  |  |  |  |

Note: MDD: Major Depressive Disorder; HC: Healthy Controls; Gain: Gain Reward; LOSS: Avoid Loss Reward.

**Table S16 Comparison of feature importance in reward magnitudes for MDD vs. HC.**

| Reward  Magnitudes | Feature Importance | (I)Feature set | (J)Feature set | 95% confidence interval | | HSD |
| --- | --- | --- | --- | --- | --- | --- |
|  | (M±SD) |  |  | lower limit | upper limit | p |
| HIGH | 0.0510 (0.0008) | HIGH | LOW | 0.0154 | 0.0158 | <.001 |
| LOW | 0.0354 (0.0008) | HIGH | CONTROL | 0.0465 | 0.0470 | <.001 |
| CONTROL | 0.0043 (0.0002) | LOW | CONTROL | 0.0309 | 0.0313 | <.001 |

Note: Reward Magnitudes: F (2, 294)=128615.675, p<.001, $\eta_{p}^{2}$=0.998; HSD: Tukey's Honest Significant Difference test. MDD: Major Depressive Disorder; HC: Healthy Controls; HIGH: High Magnitude Reward; LOW: Low Magnitude Reward; CONTROL: No Reward.

**Table S17 Comparison of feature importance in reward consequences * magnitudes for MDD vs. HC.**

| Consequences  *Magnitudes | Feature Importance | (I)Feature set | (J)Feature set | 95% confidence interval | | HSD |
| --- | --- | --- | --- | --- | --- | --- |
|  | (M±SD) |  |  | lower limit | upper limit | p |
| GH | 0.0415 (0.0018) | GH | GL | 0.0031 | 0.0042 | <.001 |
| GL | 0.0379 (0.0011) | GH | GC | 0.0365 | 0.0375 | <.001 |
| GC | 0.0045 (0.0003) | GH | LH | -0.0196 | -0.0185 | <.001 |
| LH | 0.0606 (0.0022) | GH | LL | 0.0080 | 0.0091 | <.001 |
| LL | 0.0230 (0.0011) | GH | LC | 0.0367 | 0.0377 | <.001 |
| LC | 0.0043 (0.0003) | GL | GC | 0.0328 | 0.0339 | <.001 |
|  |  | GL | LH | -0.0232 | -0.0221 | <.001 |
|  |  | GL | LL | 0.0044 | 0.0054 | <.001 |
|  |  | GL | LC | 0.0330 | 0.0341 | <.001 |
|  |  | GC | LH | -0.0566 | -0.0555 | <.001 |
|  |  | GC | LL | -0.0290 | -0.0279 | <.001 |
|  |  | GC | LC | -0.0003 | 0.0007 | 0.914 |
|  |  | LH | LL | 0.0271 | 0.0281 | <.001 |
|  |  | LH | LC | 0.0557 | 0.0568 | <.001 |
|  |  | LL | LC | 0.0281 | 0.0292 | <.001 |

Note: Reward Consequences*Magnitudes: F (5, 588)=28361.939, p<.001, $\eta_{p}^{2}$=0.996; HSD: Tukey's Honest Significant Difference test; MDD: Major Depressive Disorder; HC: Healthy Controls; GH: Gaining High (Reward); GL: Gaining Low (Reward); GC: Gaining Control (Zero Reward); LH: Losing High (Reward); LL: Losing Low (Reward); LC: Losing Control (Zero Reward).

**Table S18 Comparison of feature importance in hedonic components for SCZ vs. HC.**

| Hedonic  Components | Feature Importance | (I)Feature set | (J)Feature set | 95% confidence interval | | HSD |
| --- | --- | --- | --- | --- | --- | --- |
|  | (M±SD) |  |  | lower limit | upper limit | p |
| MOTI | 0.2077 (0.0081) | MOTI | FEEL | 0.1892 | 0.1920 | <.001 |
| FEEL | 0.0171 (0.0087) | MOTI | PRED | 0.1848 | 0.1877 | <.001 |
| PRED | 0.0214 (0.0008) | MOTI | CONS | 0.1646 | 0.1674 | <.001 |
| CONS | 0.0417 (0.0015) | MOTI | RECA | 0.1974 | 0.2002 | <.001 |
| RECA | 0.0089 (0.0004) | FEEL | PRED | -0.0057 | -0.0029 | <.001 |
|  |  | FEEL | CONS | -0.0260 | -0.0232 | <.001 |
|  |  | FEEL | RECA | 0.0068 | 0.0096 | <.001 |
|  |  | PRED | CONS | -0.0217 | -0.0189 | <.001 |
|  |  | PRED | RECA | 0.0111 | 0.0139 | <.001 |
|  |  | CONS | RECA | 0.0314 | 0.0342 | <.001 |

Note: Hedonic Components: F (4, 495)=49776.762, p<.001, $\eta_{p}^{2}$=.999; HSD: Tukey's Honest Significant Difference test; SCZ: Schizophrenia; HC: Healthy Controls; FEEL: Feeling; PRED: Prediction; CONS: Consummatory pleasure; RECA: Remembered pleasure; MOTI: Motivation.

**Table S19 Comparison of feature importance in reward consequences for SCZ vs. HC**

| Reward  Consequences | Feature Importance | Cohen d 95% confidence interval | | t | p |
| --- | --- | --- | --- | --- | --- |
|  | (M±SD) | lower limit | upper limit |  |  |
| GAIN | 0.0270 (0.0023) | -8.174 | -6.623 | -52.586 | <.001 |
| LOSS | 0.0399 (0.0010) |  |  |  |  |

Note: SCZ: Schizophrenia; HC: Healthy Controls;

**Table S20 Comparison of feature importance in reward magnitudes for SCZ vs. HC.**

| Reward  Magnitudes | Feature Importance | (I)Feature set | (J)Feature set | 95% confidence interval | | HSD |
| --- | --- | --- | --- | --- | --- | --- |
|  | (M±SD) |  |  | lower limit | upper limit | p |
| HIGH | 0.0593 (0.0013) | HIGH | LOW | 0.0299 | 0.0305 | <.001 |
| LOW | 0.0291 (0.0006) | HIGH | CONTROL | 0.0481 | 0.0487 | <.001 |
| CONTROL | 0.0109 (0.0004) | LOW | CONTROL | 0.0180 | 0.0185 | <.001 |

Note: Reward Magnitudes: F (2, 297)=80048.145, p<.001, $\eta_{p}^{2}$=1.000; HSD: Tukey's Honest Significant Difference test; SCZ: Schizophrenia; HC: Healthy Controls; HIGH: High Magnitude Reward; LOW: Low Magnitude Reward; CONTROL: No Reward.

**Table S21 Comparison of feature importance in reward consequences * magnitudes for SCZ vs. HC.**

| Reward  Consequences | Feature Importance | (I)Feature set | (J)Feature set | 95% confidence interval | | HSD |
| --- | --- | --- | --- | --- | --- | --- |
|  | (M±SD) |  |  | lower limit | upper limit | p |
| GH | 0.0378 (0.0014) | GH | GL | 0.0063 | 0.0074 | <.001 |
| GL | 0.0309 (0.0009) | GH | GC | 0.0269 | 0.0280 | <.001 |
| GC | 0.0103 (0.0005) | GH | LH | -0.0436 | -0.0425 | <.001 |
| LH | 0.0809 (0.0026) | GH | LL | 0.0099 | 0.0110 | <.001 |
| LL | 0.0273 (0.0007) | GH | LC | 0.0258 | 0.0269 | <.001 |
| LC | 0.0114 (0.0008) | GL | GC | 0.0200 | 0.0211 | <.001 |
|  |  | GL | LH | -0.0505 | -0.0494 | <.001 |
|  |  | GL | LL | 0.0030 | 0.0041 | <.001 |
|  |  | GL | LC | 0.0189 | 0.0200 | <.001 |
|  |  | GC | LH | -0.0711 | -0.0700 | <.001 |
|  |  | GC | LL | -0.0175 | -0.0165 | <.001 |
|  |  | GC | LC | -0.0016 | -0.0006 | <.001 |
|  |  | LH | LL | 0.0530 | 0.0541 | <.001 |
|  |  | LH | LC | 0.0689 | 0.0700 | <.001 |
|  |  | LL | LC | 0.0154 | 0.0164 | <.001 |

Note: Reward Consequences*Magnitudes: F (5, 594)=36326.276, p<.001, $\eta_{p}^{2}$=0.998; HSD: Tukey's Honest Significant Difference test; GH: Gaining High (Reward); GL: Gaining Low (Reward); GC: Gaining Control (Zero Reward); LH: Losing High (Reward); LL: Losing Low (Reward); LC: Losing Control (Zero Reward).

**Table S22 Comparison of feature set importance between MDD vs. HC and SCZ vs. HC**

| Reward  Components | Feature Importance (M±SD) | | Cohen d 95% confidence interval | | t | p |
| --- | --- | --- | --- | --- | --- | --- |
|  | MDD vs. HC | SCZ vs. HC | lower limit | upper limit |  |  |
| FEEL | 0.0485 (0.0023) | 0.0171 (0.0087) | 16.514 | 20.146 | 130.269 | <.001 |
| PRED | 0.0140 (0.0005) | 0.0214 (0.0008) | -12.342 | -10.078 | -79.672 | <.001 |

Note: FEEL: Feeling; PRED: Prediction; MDD: Major Depressive Disorder; SCZ: Schizophrenia; HC: Healthy Controls.

**Table S23 Feature set importance across additional algorithms for MDD vs. HC**

| Feature  Category | Component Label | STACKING | Gini | Permutation | Feature Coverage Gain |
| --- | --- | --- | --- | --- | --- |
|  |  | (SHAP) | (RF) | (SVM) | (GBT) |
| Reward  Component | MOTI | 0.1347 | 0.0316 | 0.0140 | 0.0320 |
|  | FEEL | 0.0485 | 0.0163 | 0.0111 | 0.0158 |
|  | PRED | 0.0140 | 0.0033 | 0.0095 | 0.0017 |
|  | CONS | 0.0448 | 0.0102 | 0.0138 | 0.0093 |
|  | RECA | 0.0134 | 0.0069 | 0.0088 | 0.0092 |
| Reward Consequences | GAIN | 0.0279 | 0.0099 | 0.0096 | 0.0114 |
|  | LOSS | 0.0326 | 0.0074 | 0.0117 | 0.0062 |
| Reward Magnitudes | HIGH | 0.0510 | 0.0189 | 0.0132 | 0.0182 |
|  | LOW | 0.0354 | 0.0060 | 0.0110 | 0.0074 |
|  | CONTROL | 0.0043 | 0.0014 | 0.0078 | 0.0007 |
| Reward Consequences * Magnitudes | GH | 0.0415 | 0.0221 | 0.0125 | 0.0250 |
|  | GL | 0.0379 | 0.0062 | 0.0094 | 0.0085 |
|  | GC | 0.0043 | 0.0013 | 0.0070 | 0.0006 |
|  | LH | 0.0606 | 0.0156 | 0.0138 | 0.0114 |
|  | LL | 0.0020 | 0.0059 | 0.0126 | 0.0063 |
|  | LC | 0.0002 | 0.0016 | 0.0086 | 0.0007 |

Note: MDD: Major Depressive Disorder; HC: Healthy Controls; FEEL: Feeling; PRED: Prediction; CONS: Consummatory pleasure; RECA: Remembered pleasure; MOTI: Motivation; HIGH: High Magnitude Reward; LOW: Low Magnitude Reward; CONTROL: No Reward; GH: Gaining High (Reward); GL: Gaining Low (Reward); GC: Gaining Control (Zero Reward); LH: Losing High (Reward); LL: Losing Low (Reward); LC: Losing Control (Zero Reward).

**Table S24 Feature set importance across additional algorithms for SCZ vs. HC.**

| Feature  Category | Component Label | STACKING  (SHAP) | Gini  (RF) | Permutation  (SVM) | Feature Coverage Gain (GBT) |
| --- | --- | --- | --- | --- | --- |
| Reward  Component | MOTI | 0.2077 | 0.0567 | 0.0093 | 0.0968 |
|  | FEEL | 0.0171 | 0.0082 | 0.0060 | 0.0005 |
|  | PRED | 0.0214 | 0.0039 | 0.0038 | 0.0032 |
|  | CONS | 0.0414 | 0.0112 | 0.0032 | 0.0103 |
|  | RECA | 0.0089 | 0.0042 | 0.0016 | 0.0015 |
| Reward Consequences | GAIN | 0.0263 | 0.0079 | 0.0027 | 0.0084 |
|  | LOSS | 0.0359 | 0.0096 | 0.0049 | 0.0091 |
| Reward Magnitudes | HIGH | 0.0533 | 0.0147 | 0.0059 | 0.0172 |
|  | LOW | 0.0291 | 0.0059 | 0.0028 | 0.0074 |
|  | CONTROL | 0.0109 | 0.0044 | 0.0027 | 0.0017 |
| Reward Consequences * Magnitudes | GH | 0.0378 | 0.0137 | 0.0054 | 0.0161 |
|  | GL | 0.0309 | 0.0057 | 0.0009 | 0.0085 |
|  | GC | 0.0103 | 0.0043 | 0.0018 | 0.0007 |
|  | LH | 0.0689 | 0.0156 | 0.0064 | 0.0183 |
|  | LL | 0.0273 | 0.0088 | 0.0048 | 0.0063 |
|  | LC | 0.0114 | 0.0045 | 0.0036 | 0.0028 |

Note: SCZ: Schizophrenia; HC: Healthy Controls; FEEL: Feeling; PRED: Prediction; CONS: Consummatory pleasure; RECA: Remembered pleasure; MOTI: Motivation; HIGH: High Magnitude Reward; LOW: Low Magnitude Reward; CONTROL: No Reward; GH: Gaining High (Reward); GL: Gaining Low (Reward); GC: Gaining Control (Zero Reward); LH: Losing High (Reward); LL: Losing Low (Reward); LC: Losing Control (Zero Reward).

**Table S25 Top 20 features ranked by importance for MDD vs. HC and SCZ vs. HC using stacking models**

| MDD vs. HC | | SCZ vs. HC | |
| --- | --- | --- | --- |
| Feature Name | Importance | Feature Name | Importance |
| CONS_CV_LH | 0.4376 | MOTI_RT_LH | 0.4877 |
| MOTI_RT_GL | 0.2860 | MOTI_RT_GL | 0.3785 |
| CONS_CV_LL | 0.2509 | CONS_CV_LL | 0.3108 |
| FEEL_AA_GH | 0.2483 | PRED_CA_NLH | 0.2640 |
| RECA_CV_NGH | 0.2431 | CONS_CV_LH | 0.2399 |
| MOTI_RT_LL | 0.1837 | MOTI_RT_GH | 0.2154 |
| FEEL_AA_LH | 0.1823 | RECA_AV_LH | 0.1239 |
| MOTI_RT_LH | 0.1822 | MOTI_RT_GC | 0.1056 |
| PRED_AV_GL | 0.1314 | PRED_CV_NLC | 0.1022 |
| MOTI_RT_GH | 0.0986 | PRED_AA_GH | 0.0966 |
| PRED_CA_GL | 0.0801 | CONS_CA_GH | 0.0898 |
| CONS_CV_NLH | 0.0704 | CONS_CV_NGH | 0.0826 |
| FEEL_AV_LH | 0.0547 | RECA_CA_NLH | 0.0763 |
| CONS_CV_NLL | 0.0538 | PRED_CV_GH | 0.0742 |
| PRED_CV_GL | 0.0536 | FEEL_AV_LL | 0.0707 |
| MOTI_RT_LC | 0.0445 | CONS_CA_NGL | 0.0656 |
| RECA_CA_NLH | 0.0404 | PRED_CA_GH | 0.0558 |
| RECA_CV_NLH | 0.0395 | MOTI_RT_LL | 0.0518 |
| CONS_CA_LH | 0.0372 | CONS_CV_GL | 0.0495 |
| CONS_CA_NGL | 0.0332 | PRED_AV_LC | 0.0459 |

Note: MDD: Major Depressive Disorder; SCZ: Schizophrenia; HC: Healthy Controls; MOTI: Motivation; FEEL: Feeling; PRED: Prediction; CONS: Consummatory pleasure; RECA: Remembered pleasure; GAIN: Gain Reward; LOSS: Avoid Loss Reward; RT: Reaction Time; Meaning of the first letter of the acronym after the first “_” in Feature name: A: Anticipatory pleasure; C: Consummatory Pleasure; Meaning of the second letter of the acronym after the first “_” in Feature name: A: Arousal; V: Valence; GH: Gaining High (Reward); GL: Gaining Low (Reward); GC: Gaining Control (Zero Reward); LH: Losing High (Reward); LL: Losing Low (Reward); LC: Losing Control (Zero Reward); NGH: No Gaining High (Reward); NGL: No Gaining Low (Reward); NGC: No Gaining Control (Zero Reward); NLH: No Losing High (Reward); NLL: No Losing Low (Reward); NLC: No Losing Control (Zero Reward).

**Table S26 Detailed performance of stacking models after feature elimination.**

| Feature set | Category | ACC | SENS | SPEC | AUC |
| --- | --- | --- | --- | --- | --- |
| MOTI | MDD vs. HC | 72.92% | 69.12% | 76.32% | 80.79% |
|  | SCZ vs. HC | 82.76% | 83.33% | 82.26% | 92.50% |
| ANTI | MDD vs. HC | 81.94% | 83.82% | 80.26% | 100% |
|  | SCZ vs. HC | 74.14% | 79.63% | 69.35% | 100% |
| LOSS | MDD vs. HC | 87.50% | 89.71% | 85.53% | 100% |
|  | SCZ vs. HC | 92.24% | 92.59% | 91.94% | 100% |
| HIGH | MDD vs. HC | 89.58% | 91.18% | 88.16% | 99.52% |
|  | SCZ vs. HC | 87.93% | 92.59% | 83.87% | 100% |
| TOP 8 co-emerging | MDD vs. HC | 83.33% | 85.29% | 89.19% | 100% |
|  | SCZ vs. HC | 90.52% | 90.74% | 90.32% | 99.79% |

Note: MDD: Major Depressive Disorder; SCZ: Schizophrenia; HC: Healthy Controls; ACC: accuracy; SENS: sensitivity; SPEC: specificity; AUC: Area Under the Receiver Operating Characteristic Curve; MOTI: Motivation; ANTI: Anticipatory Pleasure; LOSS: Loss; HIGH: High: High Magnitude Reward.

**Figure S1 the ACC and AUC Learning Curve of the RF for MDD vs. HC and SCZ vs. HC**

**
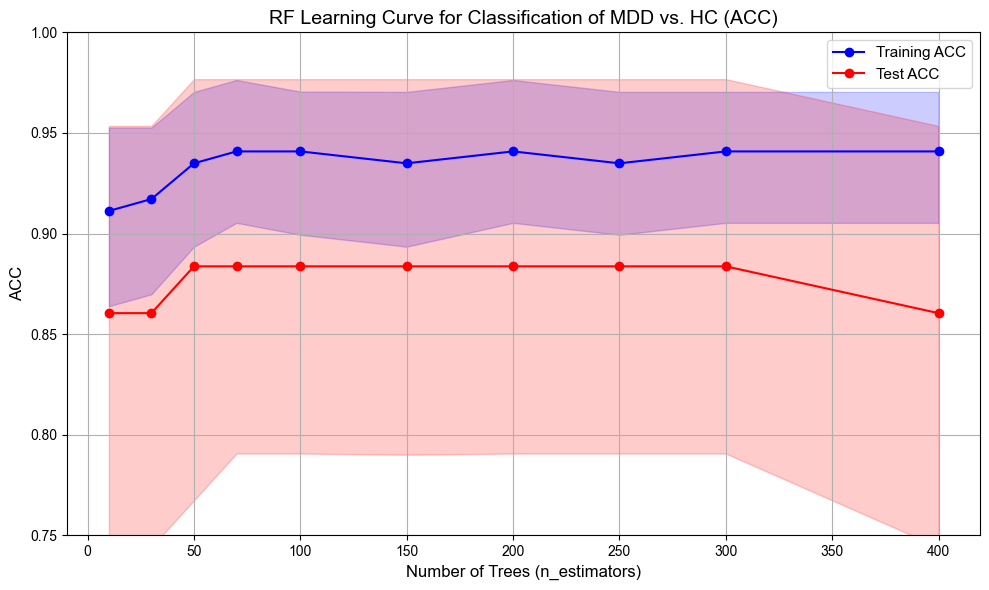

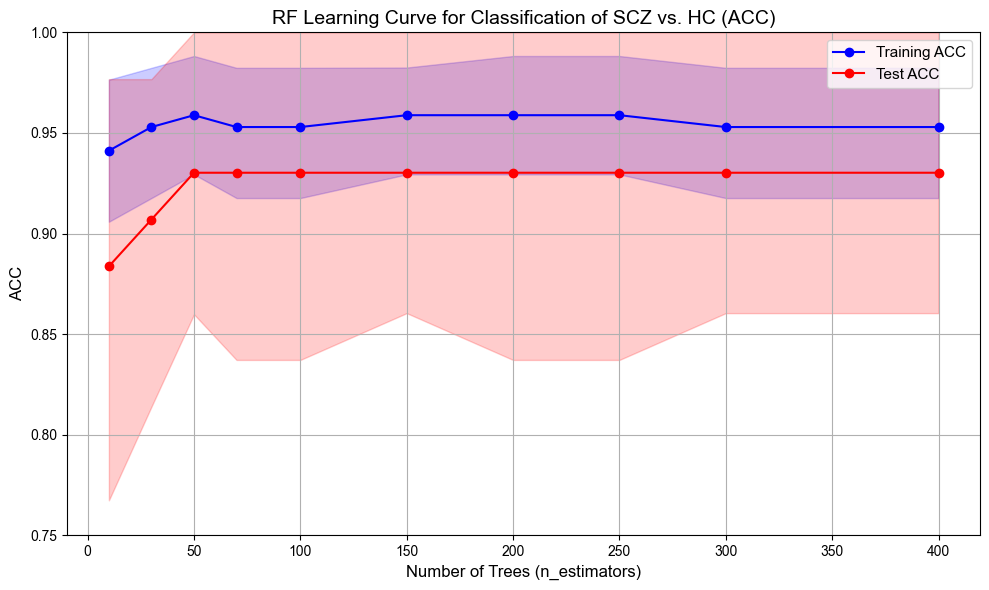
**

**
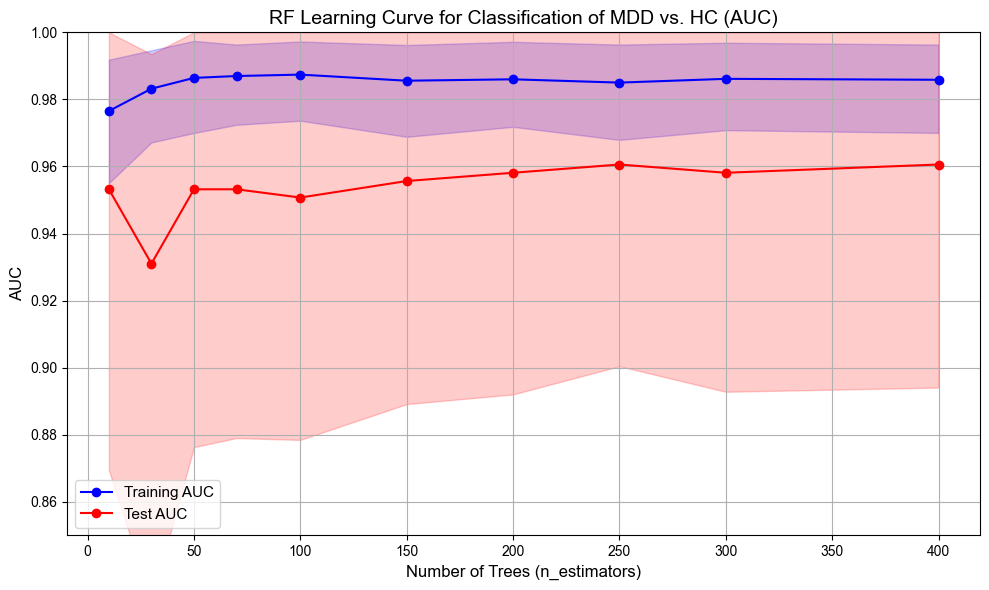

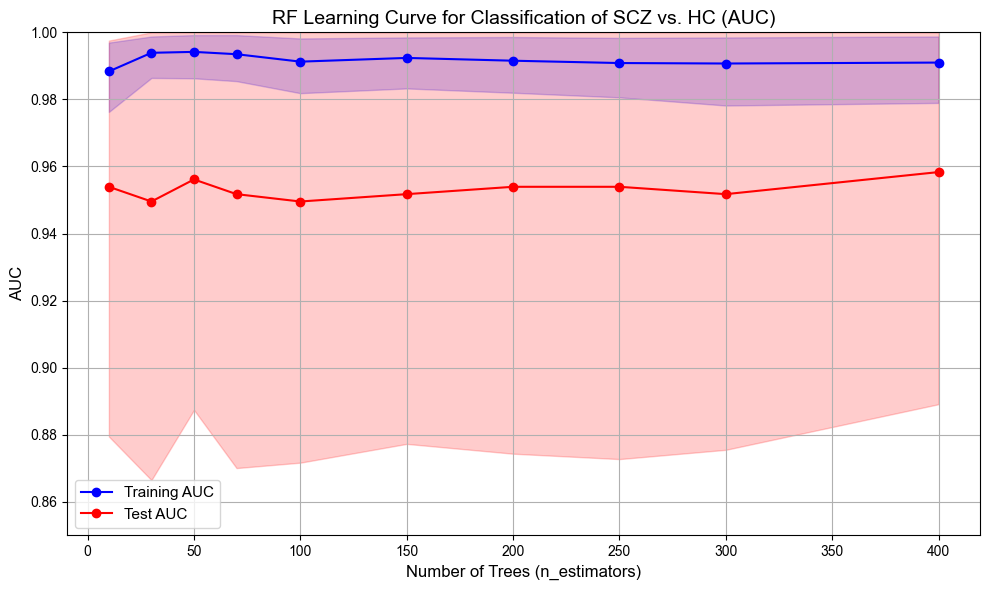
**

Note: MDD: Major Depressive Disorder; SCZ: Schizophrenia; HC: Healthy Controls; ACC: accuracy; SENS: sensitivity; SPEC: specificity; AUC: Area Under the Receiver Operating Characteristic Curve; RF: Random Forest.

**Figure S2 the ACC and AUC Learning Curve of the XGB for MDD vs. HC and SCZ vs. HC**


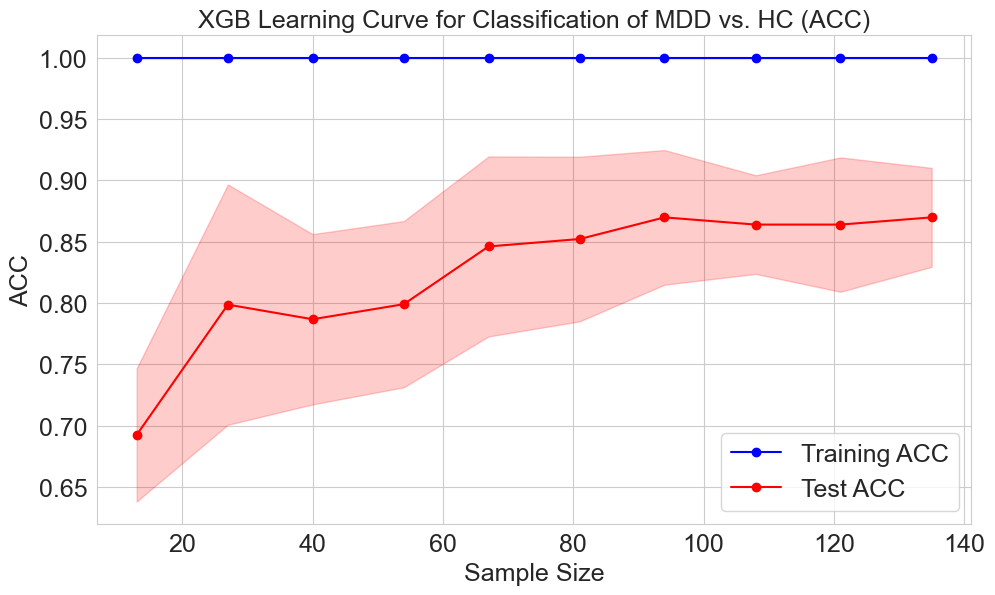

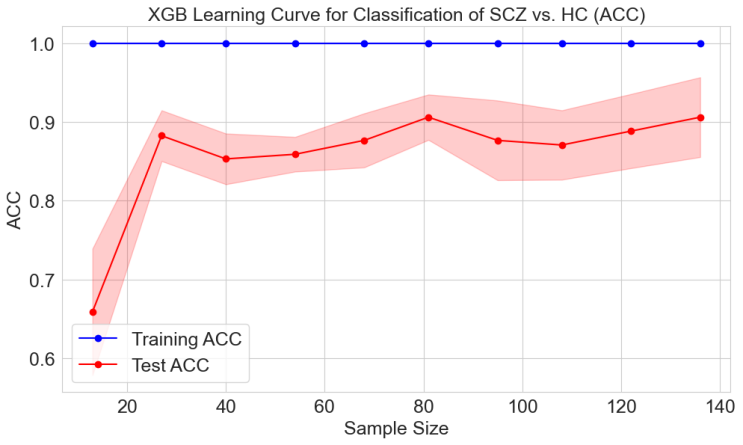


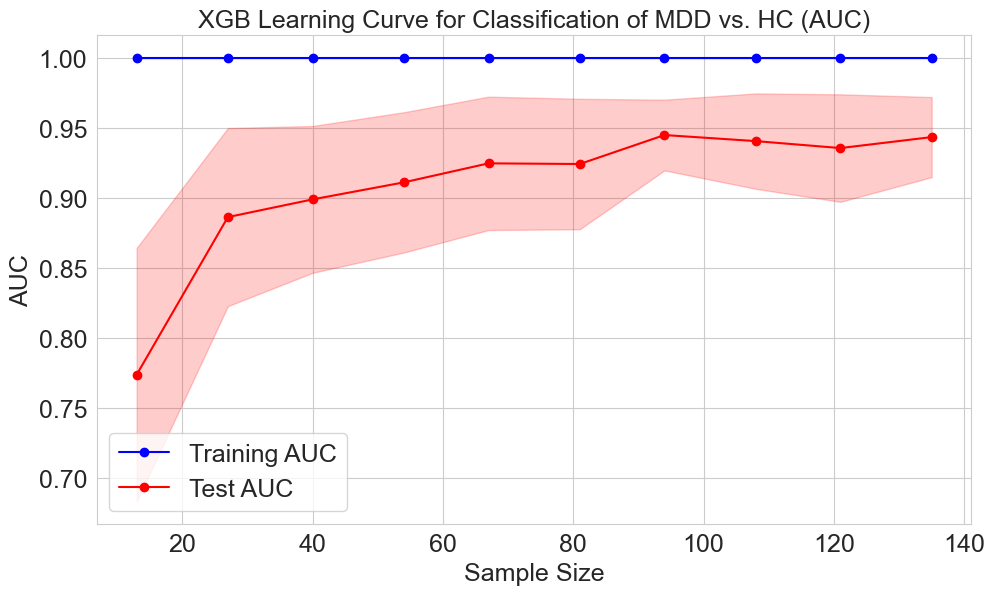

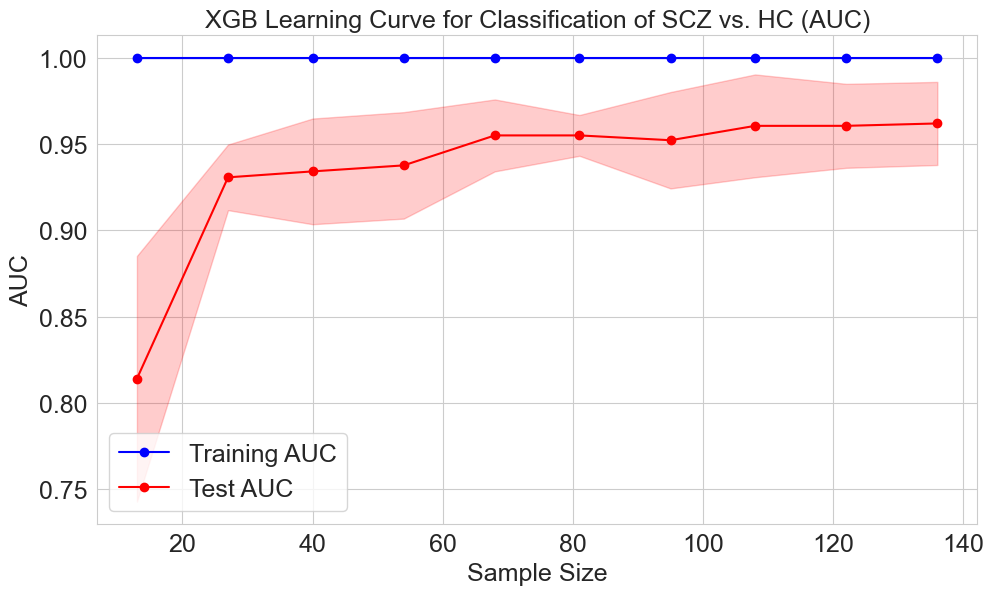


Note: MDD: Major Depressive Disorder; SCZ: Schizophrenia; HC: Healthy Controls; ACC: accuracy; SENS: sensitivity; SPEC: specificity; AUC: Area Under the Receiver Operating Characteristic Curve; XGB: eXtreme Gradient Boosting.

**Figure S3 Figure Confusion Matrix of MDD vs. SCZ**


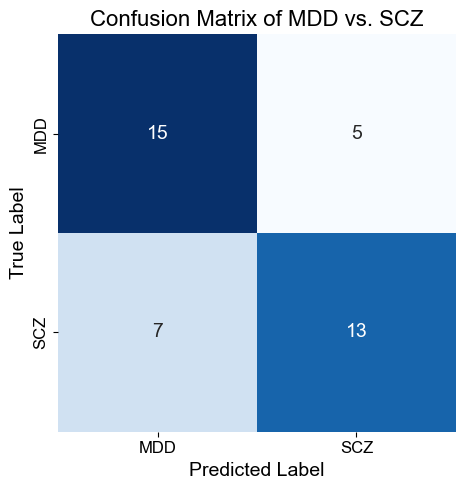

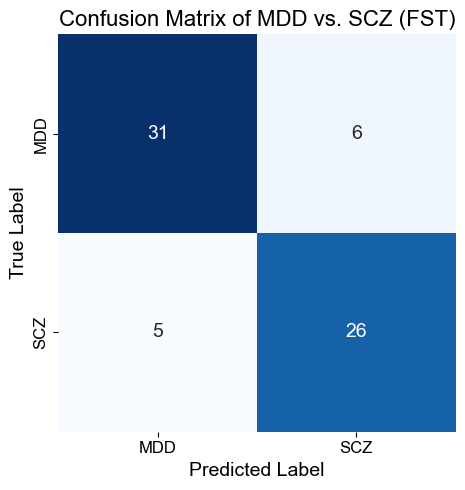


Note: MDD: Major Depressive Disorder; SCZ: Schizophrenia; FST: Fixed site training.

**Figure S4 SHAP Plot for MOTI in MDD and SCZ**


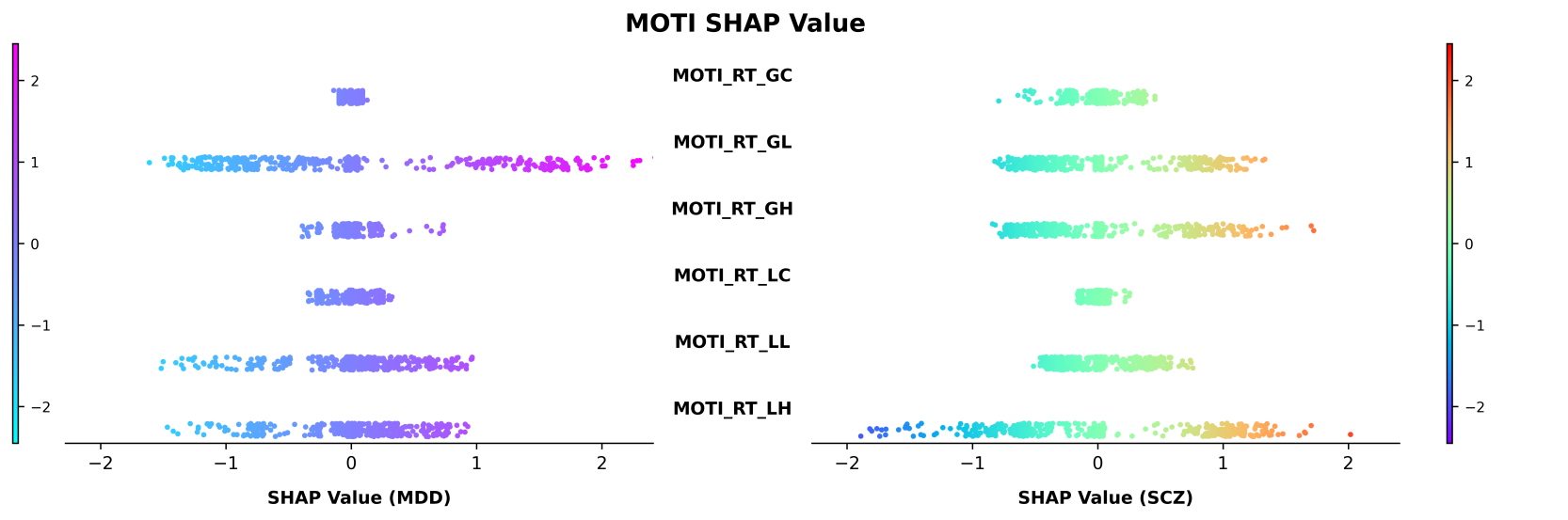
Note: MOTI: Motivation; Meaning of the second letter of the acronym after the first “_” in Feature name: A: Arousal; V: Valence; GH: Gaining High Reward; GL: Gaining Low Reward; GC: Gaining Control (Zero Rewards); LH: Losing High Reward; LL: Losing Low Reward; LC: Losing Control (Zero Rewards); NGH: No Gaining High (Reward); NGL: No Gaining Low (Reward); NGC: No Gaining Control (Zero Reward); NLH: No Losing High (Reward); NLL: No Losing Low (Reward); NLC: No Losing Control (Zero Reward).

**Figure S5 SHAP Plot for FEEL in MDD and SCZ**


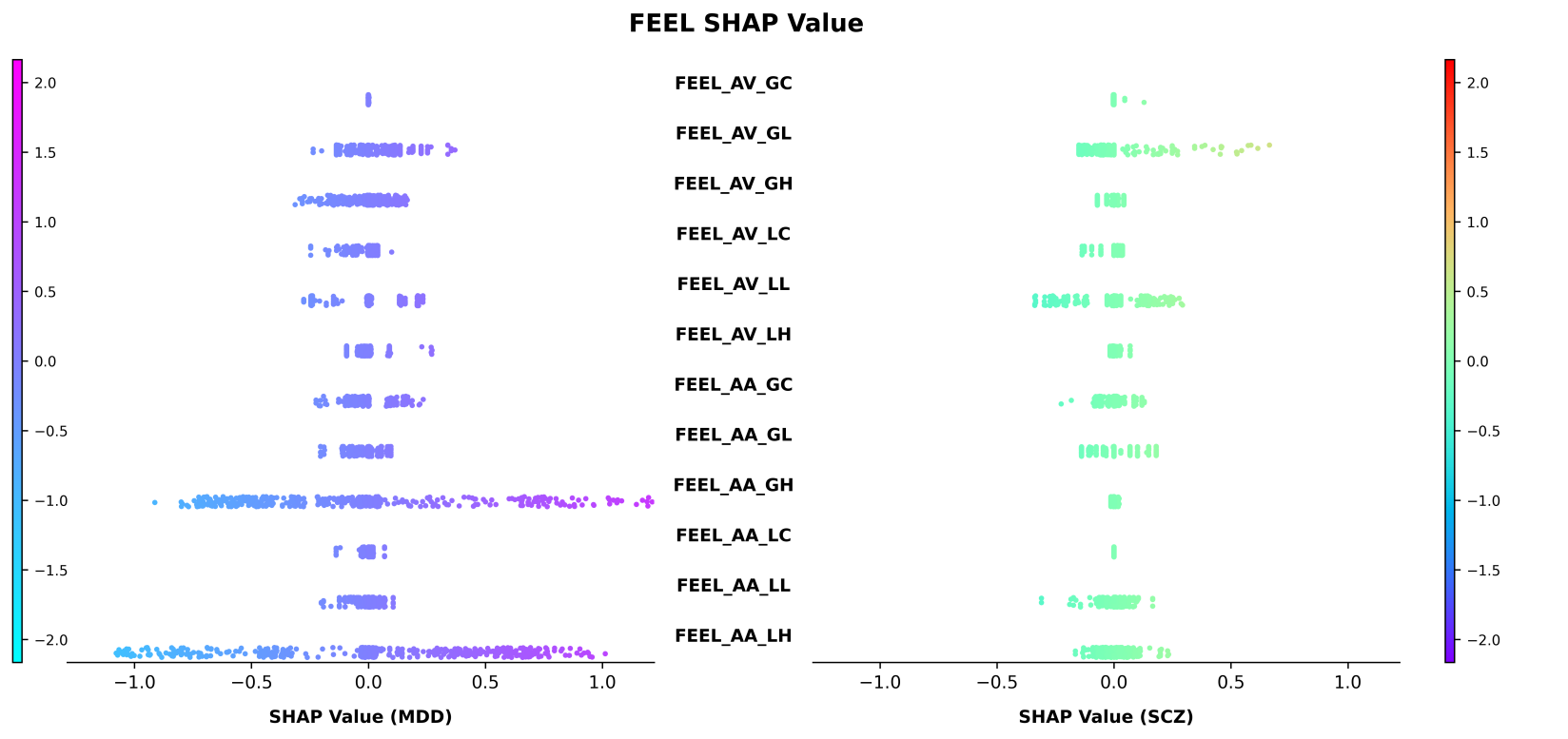
Note: FEEL: Feeling; Meaning of the second letter of the acronym after the first “_” in Feature name: A: Arousal; V: Valence; GH: Gaining High Reward; GL: Gaining Low Reward; GC: Gaining Control (Zero Rewards); LH: Losing High Reward; LL: Losing Low Reward; LC: Losing Control (Zero Rewards); NGH: No Gaining High (Reward); NGL: No Gaining Low (Reward); NGC: No Gaining Control (Zero Reward); NLH: No Losing High (Reward); NLL: No Losing Low (Reward); NLC: No Losing Control (Zero Reward).

**Figure S6 SHAP Plot for PRED in MDD and SCZ**

**
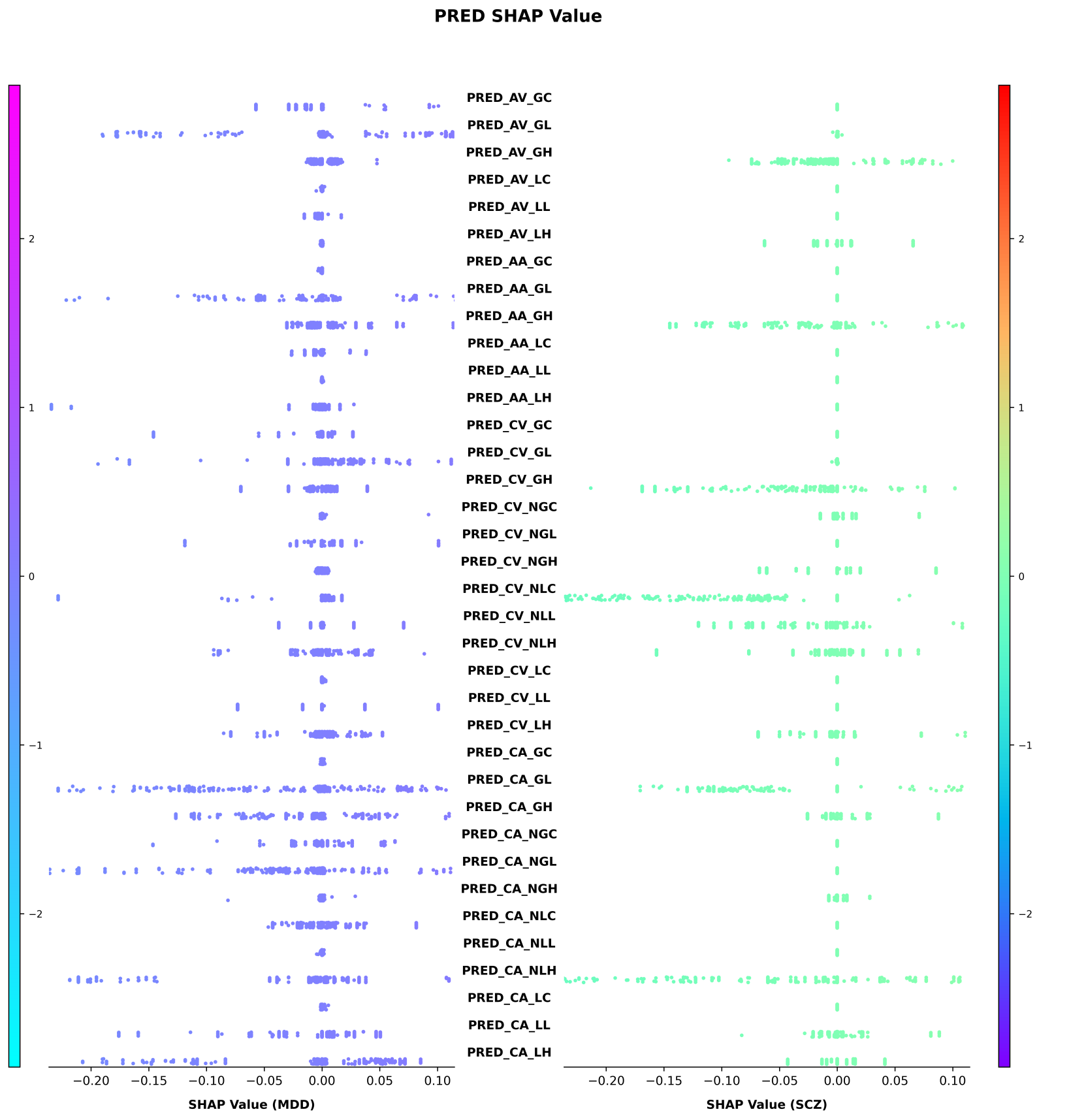
**Note: PRED: Prediction; Meaning of the second letter of the acronym after the first “_” in Feature name: A: Arousal; V: Valence; GH: Gaining High Reward; GL: Gaining Low Reward; GC: Gaining Control (Zero Rewards); LH: Losing High Reward; LL: Losing Low Reward; LC: Losing Control (Zero Rewards); NGH: No Gaining High (Reward); NGL: No Gaining Low (Reward); NGC: No Gaining Control (Zero Reward); NLH: No Losing High (Reward); NLL: No Losing Low (Reward); NLC: No Losing Control (Zero Reward).

**Figure S7 SHAP Plot for CONS in MDD and SCZ**


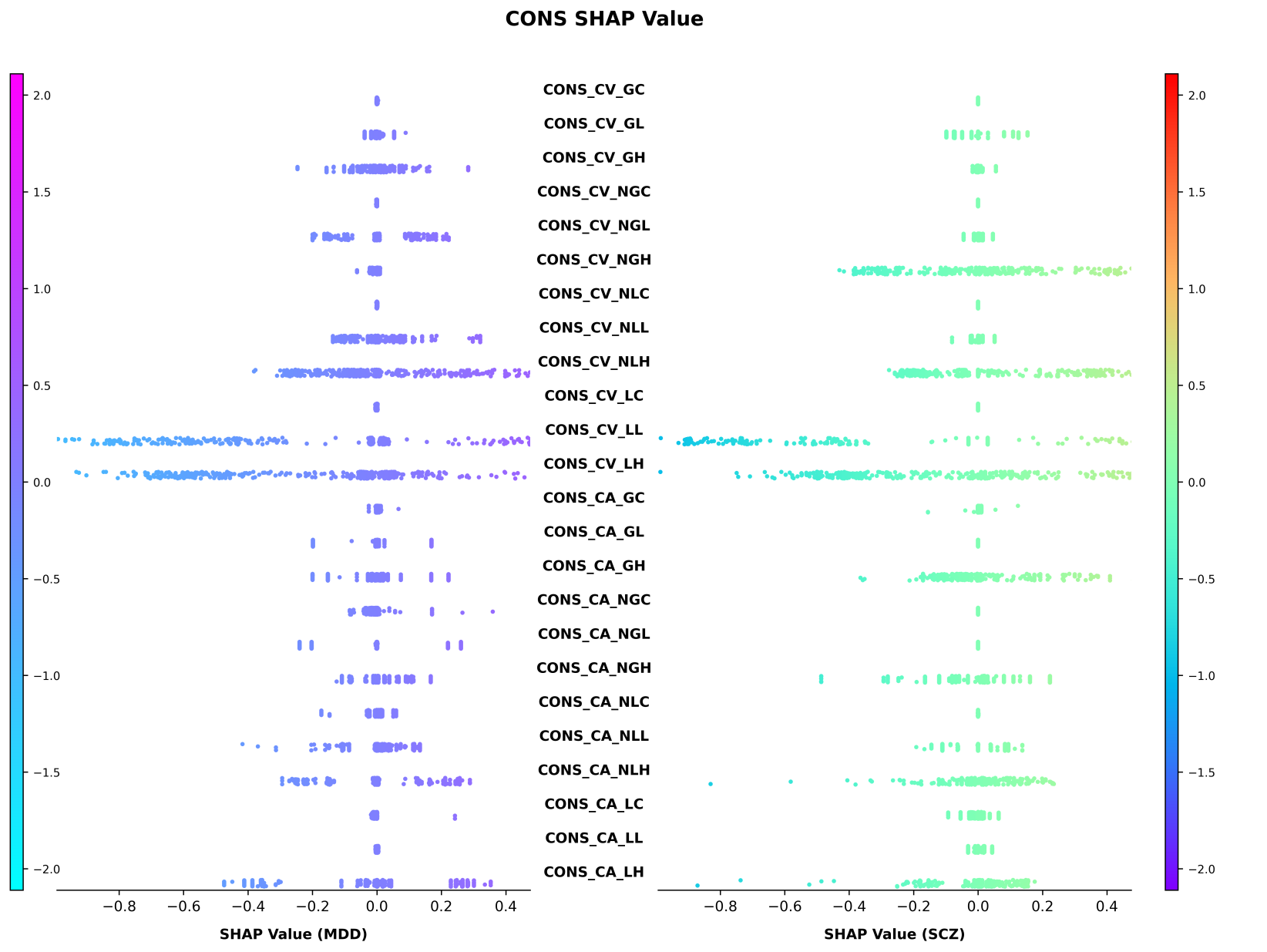
Note: CONS: Consummatory Pleasure; Meaning of the second letter of the acronym after the first “_” in Feature name: A: Arousal; V: Valence; GH: Gaining High Reward; GL: Gaining Low Reward; GC: Gaining Control (Zero Rewards); LH: Losing High Reward; LL: Losing Low Reward; LC: Losing Control (Zero Rewards); NGH: No Gaining High (Reward); NGL: No Gaining Low (Reward); NGC: No Gaining Control (Zero Reward); NLH: No Losing High (Reward); NLL: No Losing Low (Reward); NLC: No Losing Control (Zero Reward).

**Figure S8 SHAP Plot for RECA in MDD and SCZ**


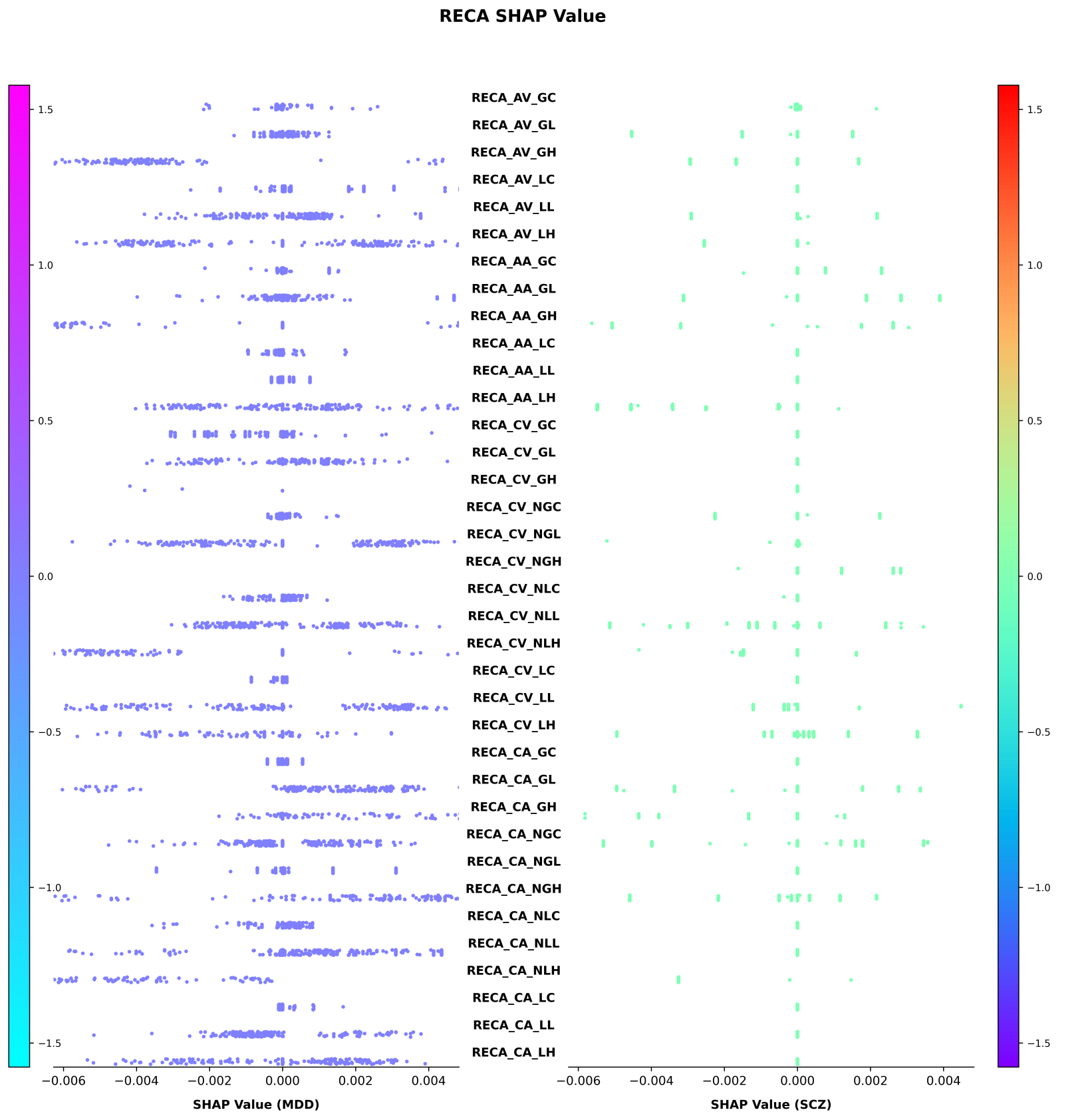
Note: RECA: Remembered Pleasure; Meaning of the second letter of the acronym after the first “_” in Feature name: A: Arousal; V: Valence; GH: Gaining High Reward; GL: Gaining Low Reward; GC: Gaining Control (Zero Rewards); LH: Losing High Reward; LL: Losing Low Reward; LC: Losing Control (Zero Rewards); NGH: No Gaining High (Reward); NGL: No Gaining Low (Reward); NGC: No Gaining Control (Zero Reward); NLH: No Losing High (Reward); NLL: No Losing Low (Reward); NLC: No Losing Control (Zero Reward).

**Note: The implementation and hyperparameter configurations of MLP and TabNet.**

Multilayer Perceptron (MLP):

MLP is a general-purpose deep learning model well-suited for capturing complex nonlinear relationships in structured data. We implemented an MLP using Keras with a TensorFlow backend, consisting of two hidden layers with 32 and 16 units, respectively, using ReLU activation, L2 regularization (λ = 1e-3), and 50% dropout. The output layer was a single sigmoid unit for binary classification. The model was trained with the Adam optimizer (learning rate = 0.001) and early stopping based on validation loss. Hyperparameters were tuned via grid search. We conducted 1,000 bootstrap evaluations with stratified 80/20 train-test splits.

TabNet:

TabNet is a deep learning architecture tailored for tabular data, leveraging sequential attention to enable interpretable feature selection and representation learning. TabNet was configured with decision and attention dimensions of 16 and five decision steps. A sparsity coefficient of 0.001 and entmax activation were used to promote interpretable, sparse feature selection. The model was trained with the Adam optimizer (learning rate = 0.02), early stopping (patience = 10, max epochs = 100), and virtual batch normalization (batch size = 16, virtual batch = 8). Hyperparameters were optimized via grid search. As with MLP, performance was evaluated using 1,000 stratified bootstrapped train-test splits.
